# Stratum corneum nanotexture feature detection using deep learning and spatial analysis: a noninvasive tool for skin barrier assessment

**DOI:** 10.1093/gigascience/giae095

**Published:** 2024-12-04

**Authors:** Jen-Hung Wang, Jorge Pereda, Ching-Wen Du, Chia-Yu Chu, Maria Oberländer Christensen, Sanja Kezic, Ivone Jakasa, Jacob P Thyssen, Sreeja Satheesh, Edwin En-Te Hwu

**Affiliations:** Department of Health Technology, Technical University of Denmark, Kongens Lyngby 2800, Denmark; Department of Health Technology, Technical University of Denmark, Kongens Lyngby 2800, Denmark; Department of Health Technology, Technical University of Denmark, Kongens Lyngby 2800, Denmark; Department of Dermatology, National Taiwan University Hospital and National Taiwan University College of Medicine, Taipei 100225, Taiwan; Department of Dermatology, National Taiwan University Hospital and National Taiwan University College of Medicine, Taipei 100225, Taiwan; Department of Dermatology, Bispebjerg and Frederiksberg Hospital (BFH), University Hospitals of Copenhagen, Copenhagen 2400, Denmark; Department of Public and Occupational Health, Amsterdam Public Health Research Institute, Amsterdam University Medical Center, Amsterdam 1105, The Netherlands; Laboratory for Analytical Chemistry, Department of Chemistry and Biochemistry, Faculty of Food Technology and Biotechnology, University of Zagreb, Zagreb 10000, Croatia; Department of Dermatology, Bispebjerg and Frederiksberg Hospital (BFH), University Hospitals of Copenhagen, Copenhagen 2400, Denmark; Institute of Solid State Physics, Leibniz University Hannover, Hannover 30167, Germany; Department of Health Technology, Technical University of Denmark, Kongens Lyngby 2800, Denmark

**Keywords:** atopic dermatitis (AD), corneocyte surface topography, deep learning, object detection, kernel density estimator (KDE), atomic force microscope (AFM)

## Abstract

**Background:**

Corneocyte surface nanoscale topography (nanotexture) has recently emerged as a potential biomarker for inflammatory skin diseases, such as atopic dermatitis (AD). This assessment method involves quantifying circular nano-size objects (CNOs) in corneocyte nanotexture images, enabling noninvasive analysis via stratum corneum (SC) tape stripping. Current approaches for identifying CNOs rely on computer vision techniques with specific geometric criteria, resulting in inaccuracies due to the susceptibility of nano-imaging techniques to environmental noise and structural occlusion on the corneocyte.

**Results:**

This study recruited 45 AD patients and 15 healthy controls, evenly divided into 4 severity groups based on their Eczema Area and Severity Index scores. Subsequently, we collected a dataset of over 1,000 corneocyte nanotexture images using our in-house high-speed dermal atomic force microscope. This dataset was utilized to train state-of-the-art deep learning object detectors for identifying CNOs. Additionally, we implemented a kernel density estimator to analyze the spatial distribution of CNOs, excluding ineffective regions with minimal CNO occurrence, such as ridges and occlusions, thereby enhancing accuracy in density calculations. After fine-tuning, our detection model achieved an overall accuracy of 91.4% in detecting CNOs.

**Conclusions:**

By integrating deep learning object detector with spatial analysis algorithms, we developed a precise methodology for calculating CNO density, termed the Effective Corneocyte Topographical Index (ECTI). The ECTI demonstrated exceptional robustness to nano-imaging artifacts and presents substantial potential for advancing AD diagnostics by effectively distinguishing between SC samples of varying AD severity and healthy controls.

## Introduction

Atopic dermatitis (AD) is a prevalent inflammatory skin disease, affecting approximately 20% of children and 5–10% of adults in high-income countries [1]. A multinational survey reported that 10–20% of adult AD patients experience severe symptoms [2]. The increasing severity of AD has been shown to significantly impact quality of life, yet reliable biomarkers for assessing disease severity are still lacking [3]. Therefore, finding an accurate measure is crucial for effective disease management and evaluating treatment efficacy.

The Eczema Area and Severity Index (EASI) [4] and SCORing AD (SCORAD) [5] scores are the commonly used clinical tools for assessing AD severity, with a preference for the EASI [6]. However, the EASI is limited by its moderate interrater reliability and a lack of interpretability data, particularly in defining the severity ranges of mild, moderate, and severe AD [7, 8]. Additionally, the EASI assigns equal weight to both extent and severity, potentially leading to a heterogeneous patient population with the same EASI score [9].

Recently, corneocyte surface nanoscale topography (nanotexture) has emerged as a potential biomarker for evaluating skin diseases, particularly through the quantification of circular nano-size objects (CNOs) in corneocyte nanotexture [10–13]. CNOs are nano-scale protrusions observed on the corneocyte surface that have been linked to skin barrier impairment [14] and AD, although their exact nature and underlying causes remain unidentified [12]. This biomarker enables noninvasive *ex vivo* analysis through stratum corneum (SC) tape stripping [15], which may serve as an objective and efficient tool for assessing AD severity.

However, the current method, known as the Dermal Texture Index (DTI), identifies CNOs in corneocyte nanotexture images by utilizing computer vision techniques that rely on specific criteria, such as height, circularity index, and area of CNOs [10, 11]. Consequently, this approach is prone to inaccuracies due to the susceptibility of nano-imaging techniques to environmental noise. Moreover, the DTI calculates CNO density across the entire corneocyte nanotexture image (20 × 20 µm^2^), which may include ineffective regions with minimal CNO occurrence, such as ridges and structural occlusions on the corneocyte surface, potentially compromising the accuracy of density calculations.

In this study, we used our in-house high-speed dermal atomic force microscope (HS-DAFM) [16] to establish an extensive database of corneocyte nanotexture images, capturing various levels of AD severity. The collected data were then leveraged to train state-of-the-art deep learning object detectors for the accurate identification of corneocyte nanotexture features. To address potential inaccuracies and artifacts arising from the nano-imaging process, we further analyzed the spatial distribution of the detected features, aiming to enhance robustness in calculating CNO density. For statistical analyses, this study investigated variations in corneocyte surface topography across different levels of AD severity, as categorized by EASI scores. The objective was to improve current clinical methods used by physicians to assess AD severity, providing a more reliable and quantifiable evaluation tool.

## Materials and Methods

### Stratum corneum sample collection

This study included a total of 45 AD patients and 15 healthy controls in Taiwan (≥18 years). Ethics approval was obtained from the National Taiwan University Hospital (202204089RIND), and all participants provided written informed consent prior to participation. The sample size was estimated using Cochran’s formula, based on a 6.7% prevalence of AD in the Taiwanese population [17], with an 80% confidence level and a 5% margin of error [18, 19]. The AD patients were evenly divided into 3 severity groups of 15 patients each, based on their EASI scores: G1 (AD mild, EASI = 0.1–7.0), G2 (AD moderate, EASI = 7.1–21.0), and G3 (AD severe, EASI > 21.0). The healthy controls were categorized as G4 (no AD history). We systematically collected SC samples from both lesional and nonlesional skin areas of each AD patient, ensuring a comprehensive representation of AD severity. No specific instructions were given regarding the interruption of topical treatment to ensure that the collected SC samples closely reflected real-world clinical scenarios. However, we acknowledge the potential influence of topical treatment at the lesional collection sites.

The SC samples were obtained using a standardized tape-stripping procedure [20]. During sampling, we collected 5 consecutive circular adhesive tape strips (D101, 1.54 cm^2^, D-Squame; Clinical & Derm) from the volar side of the forearm, approximately 10 cm below the elbow crease. Each tape strip was pressed onto the skin for 10 seconds using a pressure instrument (D500, D-Squame; Clinical & Derm) to maintain a constant pressure of 225 g/cm^2^. Subsequently, we gently removed each tape strip with tweezers and stored them individually in sampling vials.

The initial 2 strips were excluded from analysis to minimize potential contamination or impurities on the skin surface. The third strip underwent RNA analysis [21], the fourth strip was used for surface topography imaging with our HS-DAFM, and the fifth strip was analyzed for natural moisturizing factors (NMFs) [22]. The SC tapes designated for atomic force microscope (AFM) topography measurement were stored at room temperature, while the remaining tapes were immediately stored at −80°C until further analysis. This study focused on analyzing corneocyte surface topography as a potential biomarker for AD severity assessment. Results from RNA and NMF analyses will be detailed in upcoming publications.

### Corneocyte surface topography dataset

To measure corneocyte nanotexture, we utilized an HS-DAFM equipped with an aluminum-coated silicon–nitride AFM probe (spring constant of 0.03 N/m, CSC38/Al; MikroMasch) with a tip radius of 8 nm. The SC samples were measured in contact mode at a constant height, with the contact force maintained below 10 nN to ensure consistent measurement quality. The HS-DAFM scanner was calibrated using a piece of DVD data track layer (approximately 1 × 1 cm^2^) as the calibration sample [23]. The DVD data tracks are characterized by a fixed period of 740 nm and a defined depth of 160 nm, allowing precise scanner calibration through their measurement.

For each SC sample, 10 random areas were selected to capture the surface topographical features of corneocytes, resulting in a comprehensive dataset of over 1,000 corneocyte nanotexture images. Each image was acquired at a resolution of 512 × 512 pixels, covering an imaging area of 20 × 20 µm^2^. The scanning range was chosen based on findings from [11], which specify the typical dimensions of CNOs (273 nm in height and 305 nm in width), ensuring that the selected area is appropriate for capturing relevant nanoscale features.

### Image preprocessing

Corneocyte nanotexture features are often challenging to discern due to the limited contrast level in AFM imaging [24] and their intricate structured backgrounds [25]. Therefore, we applied a series of image-processing techniques to enhance the visibility of minute features, such as CNOs, while effectively suppressing environmental noise. This enhancement facilitated the subsequent process of image annotation and CNO detection.

Initially, we applied Gaussian filtering to smooth the raw images [26], followed by subtracting the mean intensity across each row to effectively mitigate striping artifacts in AFM imaging [27, 28]. Subsequently, the images were normalized to a range of 0.0 to 1.0 to ensure consistent intensity levels across all samples. Finally, disk-shaped morphological elements, with diameters of 9 and 15 pixels, were applied as percentile filters, systematically scanning the entire image to enhance local contrast and improve the visibility of subtle features, such as CNOs [29–31].

**Figure 1:**
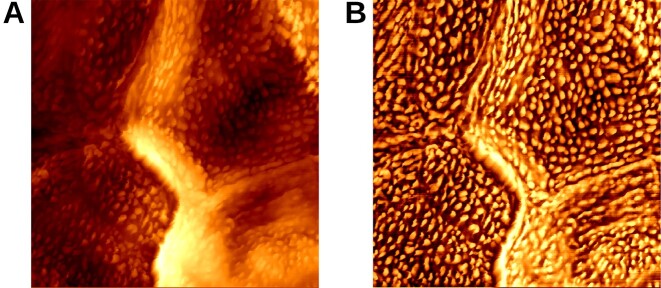
Demonstration of a corneocyte nanotexture image before and after applying the image enhancement algorithms. (A) Original corneocyte nanotexture image captured using HS-DAFM. (B) Enhanced image revealing clearer CNO contours.

Figure 1 shows the result of the image enhancement algorithms, demonstrating improved visibility of CNOs in a corneocyte nanotexture image captured from an SC sample of an AD patient.

### Training deep learning object detectors for CNO detection

Object detection is a critical task in computer vision that involves identifying and localizing objects within an image, and it has become a widely used technology in fields ranging from autonomous driving [32, 33] to medical imaging [34, 35]. In this study, we evaluated the performance of 2 state-of-the-art deep learning object detection approaches—convolutional neural network (CNN)-based detectors [36–40] and transformer-based detectors [41–47]—specifically for identifying CNOs in corneocyte nanotexture images.

Among CNN-based models, the YOLO (You Only Look Once) series [38–40, 48–58] has emerged as the most popular framework for real-time object detection, renowned for its optimal balance between speed and accuracy [59–61]. The latest iteration, YOLOv10 [58], introduces notable advancements, such as nonmaximum suppression (NMS)–free training and large-kernel convolutions, which enhance its efficiency and accuracy, particularly in the detection of small, intricate features [62, 63]. In contrast, transformer-based detectors enable end-to-end object detection [64] by employing self-attention mechanisms, which eliminate the need for NMS postprocessing. Building on this framework, RT-DETR (Real-Time Detection Transformer) [65, 66] further implements an efficient hybrid encoder and introduces uncertainty-minimal query selection to improve both accuracy and latency.

To train the object detectors, we systematically selected a dataset of 300 corneocyte nanotexture images with diverse AD severities. Each image was meticulously labeled, contributing a comprehensive dataset with an average of approximately 250 annotated CNOs per image and over 74,000 annotations in total. The dataset was then randomly split into 3 subsets for training and evaluating the object detectors: an 80% training set, a 10% validation set, and a 10% test set. Additionally, we applied a range of data augmentation techniques [67, 68] to expand the training set 3-fold, including adjustments to brightness (−25% to 25%), exposure (−15% to 15%), blur (up to 1 pixel), noise (up to 2% of pixels), and Mosaic augmentation [48].

In this study, we focused on fine-tuning YOLOv10 and RT-DETRv2 [66] models for CNO detection using our corneocyte nanotexture image dataset. Specifically, we compared the performance of various scales within each model—namely, YOLOv10-{N, S, M, B, L, X} and RT-DETRv2-{S, M, L, X}—to determine the optimal configuration for CNO detection. All models were trained and evaluated on an NVIDIA Tesla T4 GPU in Google Colab, following the same train-from-scratch settings as in [58, 65], respectively. Due to computational limitations, we adjusted the batch size as necessary. Detailed hyperparameter settings for each model are provided in Supplementary Tables S1 and S2 for further reference.

### Spatial analysis using kernel density estimator

The calculation of CNO density can exhibit significant variability due to the high sensitivity of nano-imaging techniques to environmental noise and structural occlusions on the corneocyte surface. Moreover, regions such as ridges or fringes on the corneocyte tend to have minimal CNO presence, which may compromise the accuracy of density calculations. This inherent variability in CNO distribution poses challenges in obtaining consistent and reliable density estimates.

To address these issues, we implemented a kernel density estimator (KDE) [69, 70] to generate a continuous, probabilistic density map that captures the spatial distribution of CNOs across the corneocyte surface. KDE provides a flexible framework to estimate densities from sparse and unevenly distributed data points, such as CNO coordinates, by smoothing the distribution over the entire surface. A critical parameter in KDE is the kernel’s bandwidth (BW), which determines the smoothness of the density estimate. An overly small BW results in undersmoothing, amplifying minor variations and noise in the data, whereas an excessively large BW oversmooths the density map, potentially obscuring important structural details.

**Figure 2:**
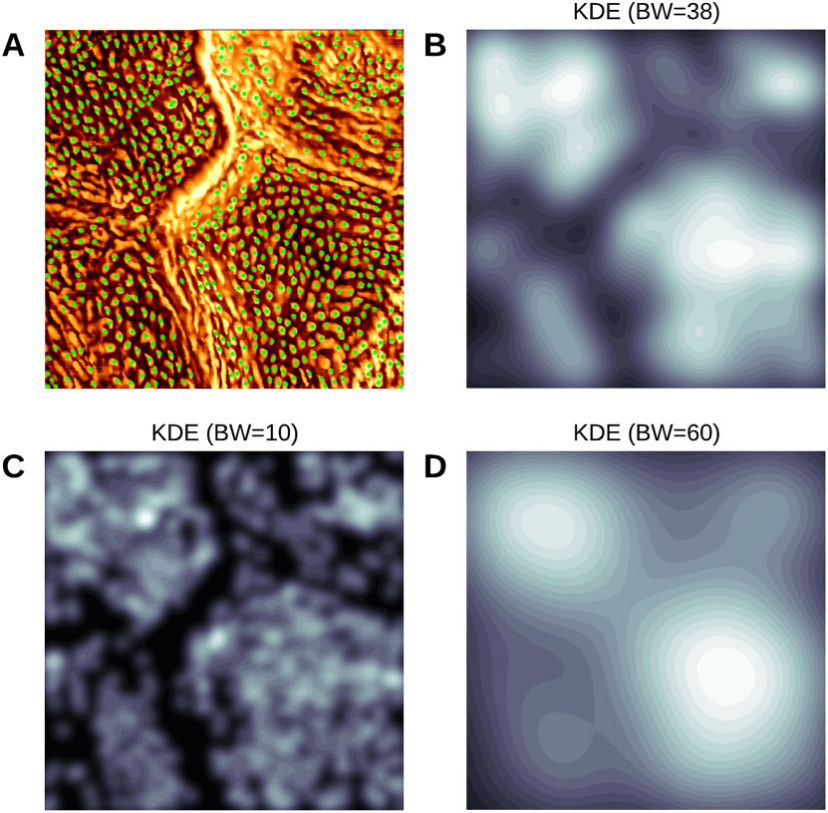
Optimal BW selection for KDE using cross-validation. (A) Corneocyte nanotexture image with detected CNOs marked as green spots. (B) Selected optimal BW = 38. (C) Example of undersmoothing (BW = 10). (D) Example of oversmoothing (BW = 60).

To optimize KDE performance, we empirically tuned the BW using cross-validation to balance between undersmoothing and oversmoothing [71]. This approach ensures that the density map accurately reflects the spatial variation in CNO distribution, while minimizing the influence of noise or occlusion artifacts. As shown in Fig. 2, the selection of BW has a substantial impact on the KDE output, where smaller BW values emphasize localized variations while larger BW values result in a more homogenized density map. Additionally, we divided the KDE density map into 25 discrete layers to enable a more detailed analysis of CNO distribution across various regions of the corneocyte. This stratified method allowed us to isolate and exclude regions affected by occlusion or artifacts, thereby improving the robustness of the analysis.

For subsequent analyses, we calculated the CNO density on the corneocyte surface by averaging the density values from the central 5 layers of the KDE density map, ensuring a more reliable representation of CNO distribution. In this study, the CNO density calculated using KDE was termed the Effective Corneocyte Topographical Index (ECTI).

## Analyses

### Comparative analysis of deep learning object detectors

In this section, we compare the performance of YOLOv10 and RT-DETRv2 models for CNO detection based on model scale, computational cost, detection accuracy, and inference speed. The standard average precision (AP) metrics [72, 73] were used to evaluate detection accuracy. AP provides a unified score by integrating metrics such as recall, precision, and intersection over union (IoU), ensuring an unbiased performance assessment. AP50 refers to the AP calculated at a fixed IoU threshold of 0.5, whereas AP50-95 represents the mean AP across uniformly sampled IoU thresholds from 0.50 to 0.95, with a step size of 0.05 [74]. The evaluation was conducted on a test set of 30 annotated corneocyte nanotexture images. In addition, latency was measured on an NVIDIA Tesla T4 GPU using TensorRT FP16 [75], with all test images resized to 512 × 512 pixels to align with the resolution of the corneocyte nanotexture images.

Table 1 presents the evaluation results of the YOLOv10 and RT-DETRv2 models, including the number of parameters, floating-point operations per second (FLOPS), AP at different IoU thresholds, and latency. Both object detectors achieve high AP50 scores above 83%; however, RT-DETRv2 exhibits lower AP50-95 scores compared to YOLOv10. The results show that YOLOv10 consistently outperforms RT-DETRv2 in detection accuracy across all model scales. Notably, the YOLOv10-L model achieves the highest accuracy, with an AP50 of 91.4% and an AP50-95 of 63.2%, exceeding the best-performing RT-DETRv2 variant (RT-DETRv2-S) with an AP50 of 87.6% and an AP50-95 of 39.6%.

**Table 1:** Performance comparison of YOLOv10 and RT-DETRv2 object detectors across various model scales. The table evaluates the models in terms of the number of parameters (M), FLOPS (G), AP50 (%), AP50-95 (%), and latency (ms).^a^

Model	# Parameter (M)	FLOPS (G)	AP50 (%)	AP50-95 (%)	Latency (ms)
YOLOv10-N	2.7	8.2	89.6	51.4	3.33
YOLOv10-S	8.0	24.4	90.8	55.5	4.58
YOLOv10-M	16.5	63.4	91.3	59.7	7.17
YOLOv10-B	20.4	97.7	91.1	62.5	7.58
YOLOv10-L	25.7	126.3	91.4	63.2	9.01
YOLOv10-X	31.6	169.8	91.2	62.9	10.95
RT-DETRv2-S	20.0	60.0	87.6	39.6	5.51
RT-DETRv2-M	31.0	100.0	84.0	37.2	7.48
RT-DETRv2-L	42.0	136.0	84.3	33.4	13.50
RT-DETRv2-X	76.0	259.0	83.3	32.0	21.15

^a^{N, S, M, B, L, X} indicate nano, small, medium, balanced, large, and extra-large models.

In terms of inference speed, both models are capable of real-time object detection. However, when comparing models of similar scales, such as YOLOv10-B with RT-DETRv2-S and YOLOv10-X with RT-DETRv2-M, RT-DETRv2 generally demonstrates lower computational costs (FLOPS) and reduced latency.

### Qualitative results

**Figure 3:**
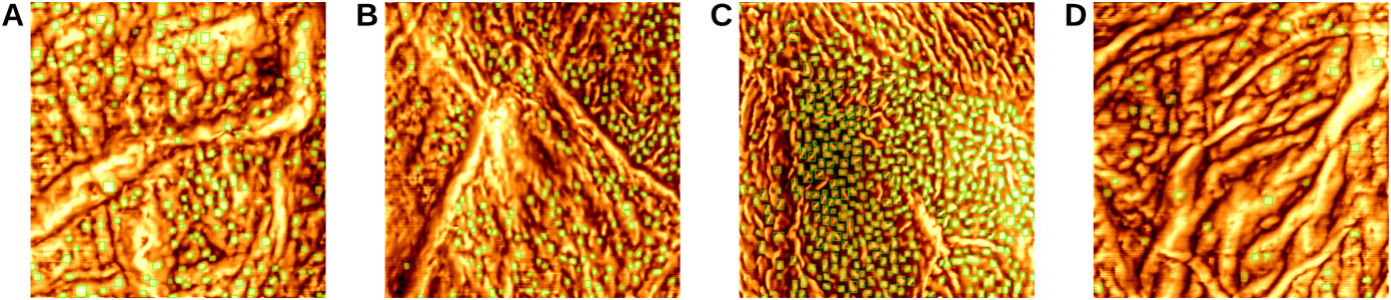
CNO detection results using YOLOv10-L model with a confidence threshold of 0.141. (A) Mild AD sample (CNO count = 180). (B) Moderate AD sample (CNO count = 250). (C) Severe AD sample (CNO count = 483). (D) Healthy control (CNO count = 22).

Figure 3 presents the qualitative results of applying the fine-tuned YOLOv10-L model to detect CNOs on corneocyte nanotexture images with different AD severity levels (G1, G2, G3, G4). The confidence threshold was set to 0.141, as this value achieved the highest F1 score [76] of 0.85, providing an optimal balance between precision and recall. The F1 confidence curve for the fine-tuned YOLOv10-L model is provided in Supplementary Fig. S2. The results demonstrate the model’s capability to accurately quantify in quantifying CNOs, even in the presence of vibrational noise introduced during topographic imaging.

**Figure 4:**
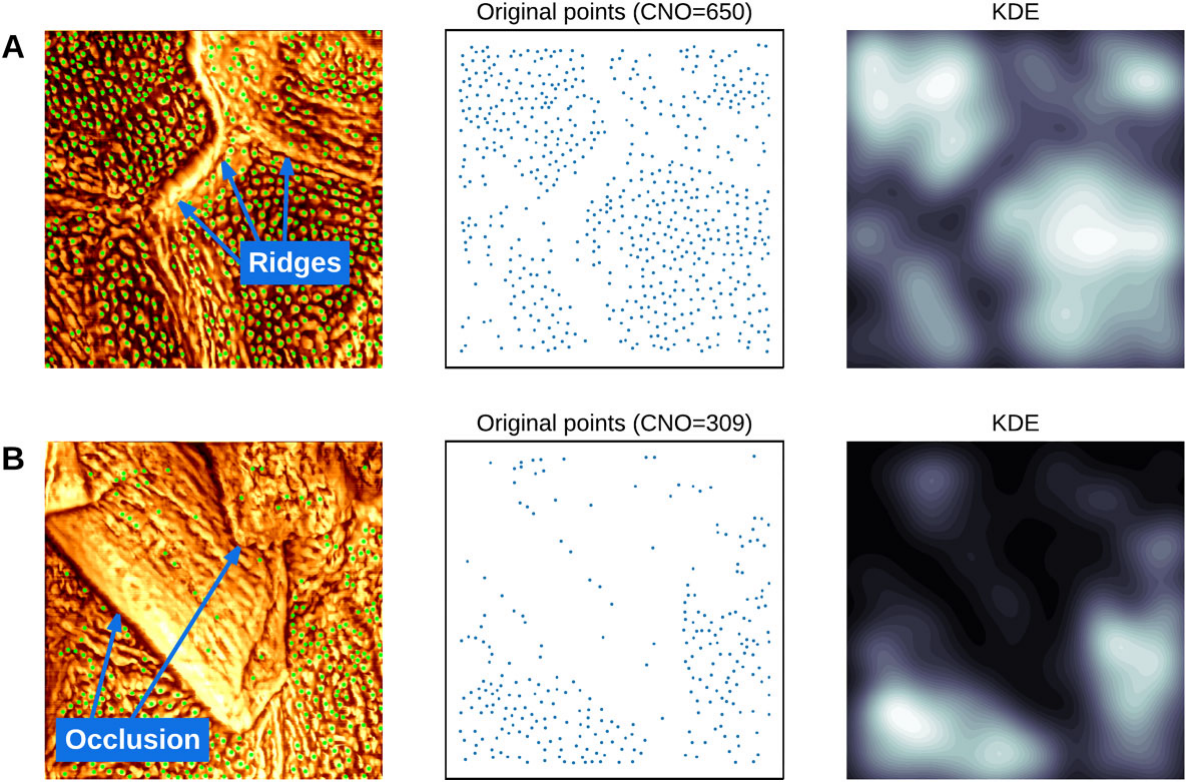
Spatial analysis of CNO distribution using KDE. (A) Corneocyte nanotexture image visualizing the presence of prominent ridges. (B) Corneocyte nanotexture image visualizing an area affected by occlusion. The KDE maps illustrate varying CNO densities, with brighter regions indicating higher densities and darker regions representing lower densities.

Figure 4 presents the analysis results of CNO distribution using KDE, in which the algorithm generates a density map representing the spatial distribution of CNOs. This process effectively excludes regions with ridges or occlusions, thereby improving the accuracy of CNO density calculations.

### Ablation study on KDE

To evaluate the impact of KDE on the variability of CNO density calculations, we conducted an ablation study, comparing results with and without KDE across different AD severity groups. The coefficient of variation (CV) [77] was used as a measure of variability, with lower CV values indicating more stable and consistent density estimates. For each SC sample, the CV was calculated from 10 density estimates derived from its corneocyte nanotexture images. The mean CV for each AD severity group was then determined by averaging the CVs of all samples within the group.

**Figure 5:**
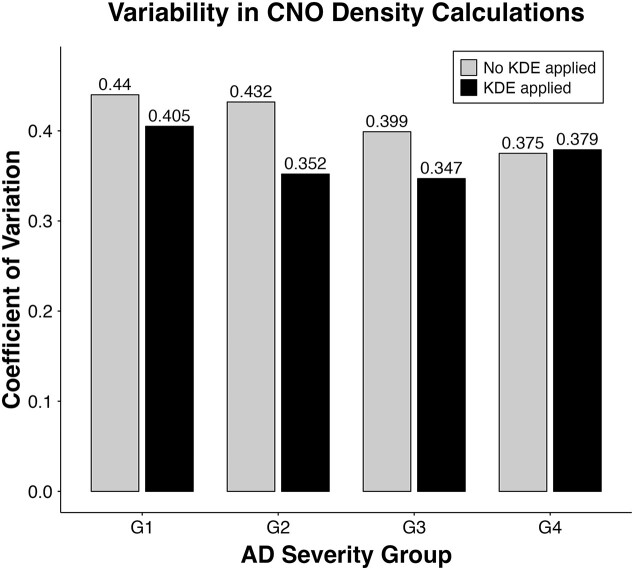
Effect of KDE on the variability in CNO density calculations across AD severity groups (G1, G2, G3, G4). The gray bars indicate CV values without KDE; the black bars show CV values with KDE applied.

As shown in Fig. 5, the application of KDE led to a notable reduction in the CV across most AD groups (G1 to G3), while G4 remained nearly unchanged. Without KDE, the CV values were consistently higher, indicating higher variability in the raw CNO density calculations. Specifically, applying KDE resulted in a reduction of 7.95% in G1 (from 0.440 to 0.405), 18.5% in G2 (from 0.432 to 0.352), 13.0% in G3 (from 0.399 to 0.347), and a slight increase of 1.1% in G4 (from 0.375 to 0.379).

The ablation study demonstrates the effectiveness of KDE in generating more robust density estimates by reducing variability, particularly in AD groups (G1 to G3) with higher CNO presence.

### Statistical analysis

The mean ECTI scores, derived from the KDE analyses of 10 corneocyte nanotexture images per SC tape, were used for statistical analyses. Each AD group (G1, G2, G3) contributed a total of 30 data points, comprising 15 from lesional and 15 from nonlesional SC samples. In contrast, the healthy control group (G4) contributed 15 data points exclusively from nonlesional SC samples. All images were preprocessed and CNOs were identified using the fine-tuned YOLOv10-L models.

Initially, samples from each AD severity group (G1, G2, G3, G4) underwent the Shapiro–Wilk normality test [78] to assess their data distribution. Given the nonnormal distribution observed in most data groups, the Wilcoxon signed-rank test [79] was adopted to determine statistically significant differences between paired samples, focusing on the comparison of lesional and nonlesional SC samples from the same AD patient. In addition, the Wilcoxon rank-sum test [80] was applied to identify significant differences between independent sample groups, specifically among the AD severity groups G1, G2, G3, and G4. Samples with missing data or those that could not be paired for comparison were excluded from the analysis.

**Figure 6:**
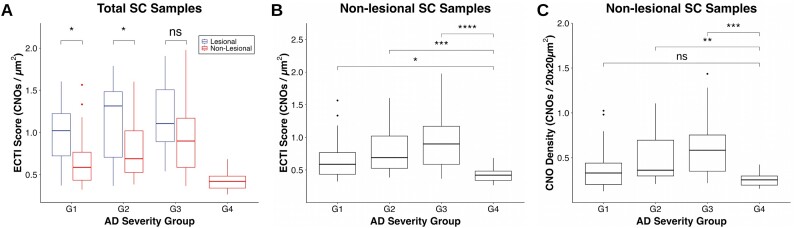
Statistical results of ECTI scores in SC samples from AD patients (*n* = 15 for both lesional and nonlesional skin areas in each group) and healthy controls (*n* = 15). (A) Comparison of ECTI scores between lesional and nonlesional SC samples across AD severity groups. (B) ECTI scores for nonlesional SC samples. (C) CNO density in nonlesional SC samples calculated over the entire imaging area (20 × 20 µm^2^) without KDE. Box plot notations: ns → not significant, *P ≤ 0.05, **P ≤ 0.01; AD severity groups according to EASI score: G1 → mild AD, G2 → moderate AD, G3 → severe AD, G4 → healthy controls.

Figure 6A presents the statistical results using box plots, further subdividing each AD severity group into lesional and nonlesional sampled areas. Overall, the plot reveals a clear trend of increasing ECTI scores corresponding to the AD severity. Most AD severity groups exhibit significant differences between lesional and nonlesional SC samples, indicating a higher occurrence of CNOs in the lesional skin areas. Additionally, the healthy controls (G4) consistently demonstrate the lowest ECTI scores compared to other AD severity groups. Figure 6B presents the statistical analysis of nonlesional SC samples across AD severity groups (G1, G2, G3) compared to the healthy control group (G4), demonstrating significant differences between the AD groups and the healthy controls.

Figure 6C provides a comparative analysis of CNO density in nonlesional SC samples calculated over the entire imaging area (20 × 20 µm^2^) without using KDE to exclude ineffective regions. The results demonstrate less significant differences between AD severity groups and the healthy control group, particularly being unable to differentiate between mild AD (G1) and healthy controls.

## Discussion

The findings of this study demonstrated the potential of corneocyte nanotexture as a reliable biomarker for assessing AD severity, particularly through CNO density calculation. By integrating state-of-the-art deep learning object detectors with spatial analysis algorithms, we proposed the ECTI, an accurate and quantifiable measure for evaluating skin barrier impairment [14]. The ECTI exhibited remarkable robustness in overcoming the inherent challenges of nano-imaging, such as environmental noise and structural occlusions on the corneocyte surface, further enhancing its applicability in clinical settings.

Previous studies revealed significant differences in corneocyte nanotexture between healthy and AD skin samples without specifying the clinical scoring of AD severity, resulting in a lack of in-depth analysis for AD severity assessment [12]. In our study, we conducted statistical analyses of ECTI scores across different AD severity groups (G1, G2, G3, G4), categorized by their EASI scores. The results revealed a clear trend of increasing ECTI scores with higher AD severity and demonstrated significant differences between AD skin samples of varying severity and healthy controls, in both lesional and nonlesional skin areas. This finding aligns with clinical observations of AD severity, offering clinicians a more objective tool for assessing the skin disease.

By leveraging deep learning object detectors, we addressed the limitations of the existing DTI method, which is prone to inaccuracies due to its dependence on fixed geometric criteria for CNO identification. To determine the optimal model architecture for CNO detection, we evaluated the performance of 2 state-of-the-art object detectors with various scales: YOLOv10-{N, S, M, B, L, X} and RT-DETRv2-{S, M, L, X}. Both models demonstrated robust performance in CNO detection, with the YOLOv10-L model achieving the highest overall accuracy (AP50) of 91.4%. Although RT-DETRv2 exhibited enhanced computational efficiency at comparable model complexities, the YOLOv10 models were more suitable for this study due to their higher detection accuracy.

Furthermore, we applied KDE to perform spatial analysis of CNO distribution. Unlike the DTI, which calculates CNO density across the entire corneocyte nanotexture image (20 × 20 ^2^ µm ) without excluding ineffective regions such as ridges and occlusions, our approach selectively excluded these areas to minimize variance in CNO density calculations. This refinement provided a more precise representation of CNO density, enabling us to effectively distinguish between mild AD (G1) and healthy controls (G4) in nonlesional SC samples.

Future work could involve expanding the corneocyte nanotexture database to include a wider range of skin diseases and conditions, providing a more comprehensive and interpretable framework for evaluating skin health through corneocyte nanotexture analysis. Additionally, integrating our findings into clinical practice could substantially improve AD severity assessment by offering an objective and quantifiable evaluation method. Clinicians could utilize corneocyte nanotexture analysis as an accessible and effective tool to monitor disease progression, assess treatment efficacy, and personalize therapeutic interventions for routine clinical use.

This study also acknowledges certain limitations. First, while the sample size in this study is adequate for preliminary analysis, it may not fully capture the variability within the broader population, particularly across diverse ethnic groups and age ranges. Second, the variability in sample collection could lead to inconsistencies. Although a standardized tape-stripping procedure was employed, variations in local eczema severity, exact sampling locations, and individual skin conditions could contribute to discrepancies in the collected SC samples. Moreover, the lack of data on factors such as emollient use, sun exposure, or bathing habits prior to sampling may further affect the results. Finally, as this study focused on AD, the applicability of our approach to other dermatological conditions remains to be validated, necessitating further research to generalize these findings to a wider range of skin diseases.

## Conclusion

This study presents a novel methodology that integrates deep learning object detection with spatial analysis to enable robust and accurate CNO density calculation within corneocyte surface topography. The ECTI was introduced as a quantifiable measure for assessing AD severity. Our results revealed significant differences in ECTI scores between SC samples of varying AD severity and healthy controls, in both lesional and nonlesional skin areas, demonstrating its potential as a reliable biomarker for AD assessment. Future work will focus on expanding the corneocyte nanotexture database and exploring the potential of ECTI in broader dermatological applications.

## Additional Files

**Supplementary Table S1**. Hyperparameter settings of YOLOv10.

**Supplementary Table S2**. Hyperparameter settings of RT-DETRv2.

**Supplementary Fig. S1**. Training results of YOLOv10-L on the corneocyte nanotexture dataset. The box loss (box) measures the error in predicted bounding box coordinates, the classification loss (cls) quantifies the error in class predictions, and distribution focal loss (dfl) adjusts the bounding box regression by focusing on more challenging examples to improve precision. “om” denotes evaluation on the training set, and “oo” indicates evaluation on the validation set.

**Supplementary Fig. S2**. F1 confidence curve of YOLOv10-L on the corneocyte nanotexture test set. This curve illustrates the relationship between the confidence threshold and the F1 score, with the highest F1 score of 0.85 achieved at a confidence threshold of 0.141. This point indicates the optimal balance between precision and recall for the model.

giae095_Supplementary_Figures_and_Tables

giae095_GIGA-D-24-00100_Original_Submission

giae095_GIGA-D-24-00100_Revision_1

giae095_Response_to_Reviewer_Comments_Original_Submission

giae095_Reviewer_1_Report_Original_Submission Xijian Fan -- 6/23/2024

giae095_Reviewer_1_Report_Revision_1 Xijian Fan -- 10/19/2024

giae095_Reviewer_2_Report_Original_Submission Zhicheng Zhang -- 7/5/2024

giae095_Reviewer_2_Report_Revision_1 Zhicheng Zhang -- 10/13/2024

## Abbreviations

AD: atopic dermatitis; AFM: atomic force microscope; AP: average precision; BW: bandwidth; CNN: convolutional neural network; CNO: circular nano-size object; CV: coefficient of variation; DTI: Dermal Texture Index; EASI: Eczema Area and Severity Index; ECTI: Effective Corneocyte Topographical Index; FLOPS: floating-point operations per second; HS-DAFM: high-speed dermal atomic force microscope; IoU: intersection over union; KDE: kernel density estimator; NMF: natural moisturizing factor; NMS: nonmaximum suppression; RT-DETR: real-time detection transformer; SC: stratum corneum; SCORAD: SCORing AD; YOLO: You Only Look Once.

## Data Availability

The corneocyte nanotexture dataset, along with the annotations used to train YOLOv10 and RT-DETRv2 object detection models, is available in the GitHub repository [81]. Fine-tuned models and source code can also be downloaded from the same repository. Workflows are archived in WorkflowHub.eu [82] and all additional supporting data and materials are accessible via the *GigaScience* database, GigaDB [83].

## Availability of Source Code and Requirements

- Project name: ECTI Atopic Dermatitis
- Project homepage: GitHub link: https://github.com/JenHungWang/ECTI_Atopic_Dermatitis
- Operating system(s): Platform independent
- Programming language: Python 3.11.4
- Other requirements: Python 3.10+, matplotlib 3.7.2, numpy 1.25.1, opencv-python 4.8.0.74, scipy 1.11.1, scikit-image 0.21.0, scikit-learn 1.3.1, ultralytics 8.2.95, customtkinter 5.2.1
- License: PSF, BSD, Apache, AGPL-3.0
- Workflowhub: https://doi.org/10.48546/workflowhub.workflow.1161.1
- ECTI is registered as a software application on SciCrunch (RRID: SCR_025706) and biotools (biotools:ecti_atopic_dermatitis)
