# Supplementary material for: Stratum corneum nanotexture feature detection using deep learning and spatial analysis: a noninvasive tool for skin barrier assessment
Source: Gigascience. 2024 Dec 4;13:giae095. doi: 10.1093/gigascience/giae095 (PMC11629979; doi:10.1093/gigascience/giae095)
Supplement: giae095_Supplementary_Figures_and_Tables [file giae095_supplementary_figures_and_tables.pdf]

Supplementary Figures and Tables for

Stratum corneum nanotexture feature detection using deep learning and spatial analysis: a non-invasive tool for skin barrier assessment

Jen-HungWang[1], Jorge Pereda[1], Ching-Wen Du[1,2], Chia-Yu Chu[2,*],Maria Oberländer Christensen[3], Sanja Kezic[4], Ivone Jakasa[5], Jacob P. Thyssen[3], Sreeja Satheesh[6] and Edwin En-Te Hwu[1,*]

[1]Department of Health Technology, Technical University of Denmark, Denmark

[2]Department of Dermatology, National Taiwan University Hospital and National Taiwan University College of Medicine, Taipei, Taiwan

[3]Department of Dermatology, Bispebjerg and Frederiksberg Hospital (BFH). University Hospitals of Copenhagen, Copenhagen, Denmark

[4]Department of Public and Occupational Health, Amsterdam Public Health Research Institute, Amsterdam University Medical Center, Amsterdam, The Netherlands

[5]Laboratory for Analytical Chemistry, Department of Chemistry and Biochemistry, Faculty of Food Technology and Biotechnology, University of Zagreb, Zagreb, Croatia

[6]Institute of Solid State Physics, Leibniz University Hannover, Hannover, Germany

[*]Correspondence address:

Chia-Yu Chu, Department of Dermatology, National Taiwan University Hospital and National Taiwan University College of Medicine, Taipei, Taiwan. E-mail: chiayu@ntu.edu.tw;

Edwin En-Te Hwu, Department of Health Technology, Technical University of Denmark, Denmark. E-mail: etehw@dtu.dk

Table S1. Hyperparameter settings of YOLOv10.

Hyperparameter	YOLOv10-N/S/M/B/L/X
epochs	800
batch size	16/16/16/16/16/8
optimizer	SGD
momentum	0.937
weight decay	5e-4
warm-up epochs	3
warm-up momentum	0.8
warm-up bias learning rate	0.1
initial learning rate	1e-2
final learning rate	1e-4
learning rate schedule	Linear decay
box loss gain	7.5
class loss gain	0.5
DFL loss gain	1.5
HSV saturation augmentation	0.7
HSV value augmentation	0.4
HSV hue augmentation	0.015
translation augmentation	0.1
scale augmentation	0.5/0.5/0.9/0.9/0.9/0.9
mosaic augmentation	1.0
Mixup augmentation	0.0/0.0/0.1/0.1/0.15/0.15
copy-paste augmentation	0.0/0.0/0.1/0.1/0.3/0.3
close mosaic epochs	10

Table S2. Hyperparameter settings of RT-DETRv2.

Hyperparameter	RT-DETRv2-S/M/L/X
epochs	120/84/72/72
batch size	8/3/3/3
optimizer	AdamW
base learning rate	1e-4
learning rate of backbone	1e-5
freezing BN	True
linear warm-up start factor	0.001
linear warm-up steps	2000
weight decay	1e-4
clip gradient norm	0.1
ema decay	0.9999
number of AIFI layer	1
number of repblocks	3
embedding dim	256
feedforward dim	1024
nheads	8
number of feature scales	3
number of decoder layers	6
number of queries	900
decoder npoints	4
Class cost weight	2.0
α in class cost	0.25
γ in class cost	2.0
bbox cost weight	5.0
GIoU cost weight	2.0
class loss weight	1.0
α in class loss	0.75
γ in class loss	2.0
bbox loss weight	5.0
GIoU loss weight	2.0
denoising number	200
label noise ratio	0.5
box noise scale	1.0

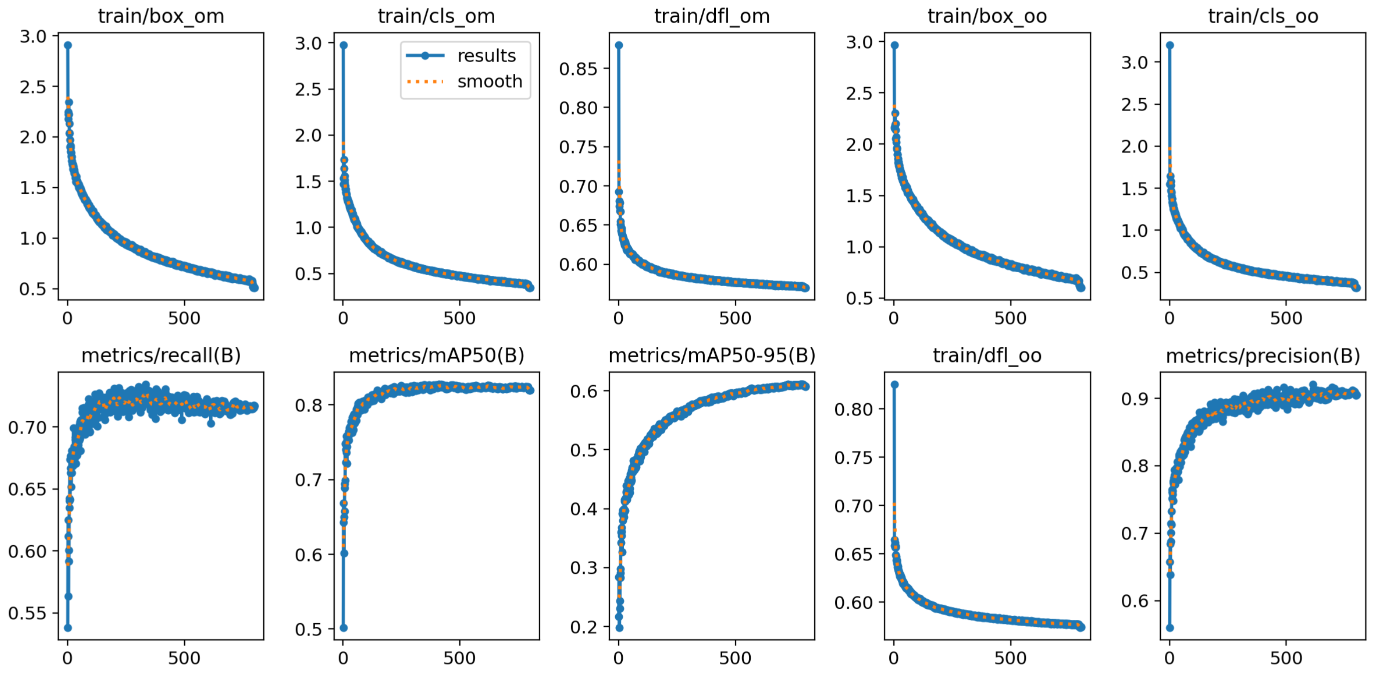

Fig. S1. Training results of YOLOv10-L on the corneocyte nanotexture dataset. The box loss (box) measures the error in predicted bounding box coordinates, the classification loss (cls) quantifies the error in class predictions, and distribution focal loss (dfl) adjusts the bounding box regression by focusing on more challenging examples to improve precision. 'om' denotes evaluation on the training set, and 'oo' indicates evaluation on the validation set.

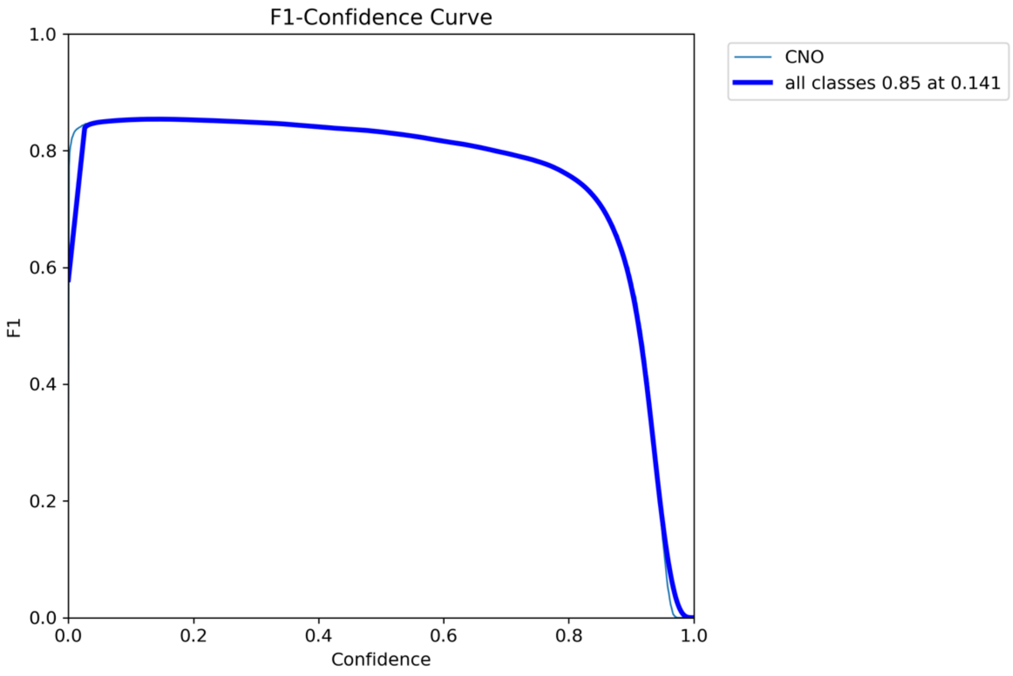

Fig. S2. F1-confidence curve of YOLOv10-L on the corneocyte nanotexture test set. This curve illustrates the relationship between confidence threshold and the F1 score, with the highest F1 score of 0.85 achieved at a confidence threshold of 0.141. This point indicates the optimal balance between precision and recall for the model.
