# Supplementary material for: Stratum corneum nanotexture feature detection using deep learning and spatial analysis: a noninvasive tool for skin barrier assessment
Source: Gigascience. 2024 Dec 4;13:giae095. doi: 10.1093/gigascience/giae095 (PMC11629979; doi:10.1093/gigascience/giae095)
Supplement: giae095_GIGA-D-24-00100_Original_Submission [file giae095_giga-d-24-00100_original_submission.pdf]

GigaScience

Stratum corneum nanotexture feature detection using deep learning and spatial analysis: a non-invasive tool for skin barrier assessment

--Manuscript Draft--

**Manuscript Number:**	GIGA-D-24-00100
**Full Title:**	Stratum corneum nanotexture feature detection using deep learning and spatial analysis: a non-invasive tool for skin barrier assessment
**Article Type:**	Research
**Funding Information:**	LEO Fondet (LF-OC-20-000370) — Mr. Edwin En-Te Hwu<br>Novo Nordisk Fonden (NNF22OC0076607) — Mr. Edwin En-Te Hwu<br>National Science and Technology Council (NSTC 112-2314-B-002-074-MY3) — Mr. Chia-Yu Chu

Abstract:

Background: Recently, corneocyte surface topography has emerged as a potential biomarker for inflammatory skin diseases, such as atopic dermatitis (AD). This evaluation approach involves quantifying circular nano-size objects (CNOs) in corneocyte nanotexture images, enabling non-invasive analysis via stratum corneum (SC) tape stripping. Current methods for identifying CNOs rely on computer vision techniques with specific geometric criteria, resulting in inaccuracies due to the susceptibility of nano-imaging techniques to environmental noise and structural occlusion on the corneocyte.

Results: This study recruited 45 AD patients and 15 healthy controls, evenly divided into four severity groups based on their Eczema Area and Severity Index (EASI) scores. Subsequently, we collected a dataset of over 1,000 corneocyte nanotexture images using our in-house high-speed dermal atomic force microscope. This dataset was utilized to train state-of-the-art deep learning object detectors for identifying CNOs. Additionally, we implemented a kernel density estimator (KDE) to analyze the spatial distribution of CNOs, excluding ineffective areas with minimal CNO occurrence, such as ridges and occlusions, thereby enhancing accuracy in density calculations. After fine-tuning, our detection model achieved an overall accuracy of 92.2% in detecting CNOs.

Conclusions: By integrating deep learning object detector with spatial analysis algorithm (KDE), we developed a precise methodology for calculating CNO density, termed the Effective Corneocyte Topographical Index (ECTI), which demonstrated exceptional robustness against nano-imaging susceptibility. The ECTI might contribute to AD diagnostics as it is able to detect differences between SC samples of varying AD severity and healthy controls.

**Corresponding Author:**	Edwin En-Te Hwu<br>Technical University of Denmark: Danmarks Tekniske Universitet<br>Kongens Lyngby, DENMARK
**Corresponding Author Secondary Information:**	
**Corresponding Author's Institution:**	Technical University of Denmark: Danmarks Tekniske Universitet
**Corresponding Author's Secondary Institution:**	
**First Author:**	Jen-Hung Wang
**First Author Secondary Information:**	
**Order of Authors:**	Jen-Hung Wang<br>Jorge Pereda<br>Ching-Wen Du<br>Chia-Yu Chu

Powered by Editorial Manager® and ProduXion Manager® from Aries Systems Corporation

	Maria Oberländer Christensen
	Sanja Kezic
	Ivone Jakasa
	Jacob P. Thyssen
	Sreeja Satheesh
	Edwin En-Te Hwu
**Order of Authors Secondary Information:**	
**Additional Information:**	
**Question**	**Response**
Are you submitting this manuscript to a special series or article collection?	No
**Experimental design and statistics**<br><br>Full details of the experimental design and statistical methods used should be given in the Methods section, as detailed in our Minimum Standards Reporting Checklist. Information essential to interpreting the data presented should be made available in the figure legends.<br><br>Have you included all the information requested in your manuscript?	Yes
**Resources**<br><br>A description of all resources used, including antibodies, cell lines, animals and software tools, with enough information to allow them to be uniquely identified, should be included in the Methods section. Authors are strongly encouraged to cite Research Resource Identifiers (RRIDs) for antibodies, model organisms and tools, where possible.<br><br>Have you included the information requested as detailed in our Minimum Standards Reporting Checklist?	Yes
**Availability of data and materials**<br><br>All datasets and code on which the	Yes

conclusions of the paper rely must be either included in your submission or deposited in publicly available repositories (where available and ethically appropriate), referencing such data using a unique identifier in the references and in the "Availability of Data and Materials" section of your manuscript.<br><br>Have you have met the above requirement as detailed in our Minimum Standards Reporting Checklist?	

Manuscript

Click here to access/download;Manuscript;Stratum corneum nanotexture feature detection.pdf

Placeholder for OUP logo oup.pdf

GigaScience, 2024, 1–7

doi: xx.xxxx/xxxx
Manuscript in Preparation
Paper

PAPER

Stratum corneum nanotexture feature detection using deep learning and spatial analysis: a non-invasive tool for skin barrier assessment

Jen-Hung Wang[1], Jorge Pereda[1], Ching-Wen Du[1,2], Chia-Yu Chu[2,*], Maria Oberländer Christensen[3], Sanja Kezic[4], Ivone Jakasa[5], Jacob P. Thyssen[3], Sreeja Satheesh[6] and Edwin En-Te Hwu[1,*]

[1]Department of Health Technology, Technical University of Denmark, Denmark and [2]Department of Dermatology, National Taiwan University Hospital and National Taiwan University College of Medicine, Taipei, Taiwan and [3]Department of Dermatology, Bispebjerg and Frederiksberg Hospital (BFH). University Hospitals of Copenhagen, Copenhagen, Denmark and [4]Department of Public and Occupational Health, Amsterdam Public Health Research Institute, Amsterdam University Medical Center, Amsterdam, The Netherlands and [5]Laboratory for Analytical Chemistry, Department of Chemistry and Biochemistry, Faculty of Food Technology and Biotechnology, University of Zagreb, Zagreb, Croatia and [6]Institute of Solid State Physics, Leibniz University Hannover, Hannover, Germany

[*]**Correspondence address.** Chia-Yu Chu, Department of Dermatology, National Taiwan University Hospital and National Taiwan University College of Medicine, Taipei, Taiwan. E-mail: chiayu@ntu.edu.tw; Edwin En-Te Hwu, Department of Health Technology, Technical University of Denmark, Denmark. E-mail: etehw@dtu.dk

Abstract

Background: Recently, corneocyte surface topography has emerged as a potential biomarker for inflammatory skin diseases, such as atopic dermatitis (AD). This evaluation approach involves quantifying circular nano-size objects (CNOs) in corneocyte nanotexture images, enabling non-invasive analysis via stratum corneum (SC) tape stripping. Current methods for identifying CNOs rely on computer vision techniques with specific geometric criteria, resulting in inaccuracies due to the susceptibility of nano-imaging techniques to environmental noise and structural occlusion on the corneocyte.
Results: This study recruited 45 AD patients and 15 healthy controls, evenly divided into four severity groups based on their Eczema Area and Severity Index (EASI) scores. Subsequently, we collected a dataset of over 1,000 corneocyte nanotexture images using our in-house high-speed dermal atomic force microscope. This dataset was utilized to train state-of-the-art deep learning object detectors for identifying CNOs. Additionally, we implemented a kernel density estimator (KDE) to analyze the spatial distribution of CNOs, excluding ineffective areas with minimal CNO occurrence, such as ridges and occlusions, thereby enhancing accuracy in density calculations. After fine-tuning, our detection model achieved an overall accuracy of 92.2% in detecting CNOs.
Conclusions: By integrating deep learning object detector with spatial analysis algorithm (KDE), we developed a precise methodology for calculating CNO density, termed the Effective Corneocyte Topographical Index (ECTI), which demonstrated exceptional robustness against nano-imaging susceptibility. The ECTI might contribute to AD diagnostics as it is able to detect differences between SC samples of varying AD severity and healthy controls.

Keywords: atopic dermatitis (AD), corneocyte surface topography, deep learning, object detection, kernel density estimation (KDE), atomic force microscope (AFM)

Compiled on: March 27, 2024.
Draft manuscript prepared by the author.

Introduction

Atopic dermatitis (AD) is a common inflammatory skin disease, affecting approximately 20% of children and 5-10% of adults in high-income countries [1]. According to a multinational survey, 10-20% of adult patients with AD reported severe symptoms [2]. Studies have observed a significant impact on quality of life with increasing AD severity, but no good biomarkers exist to measure severity [3]. Therefore, searching for a reliable measure to assess AD severity is essential for disease management and evaluating the treatment effectiveness. Eczema Area and Severity Index (EASI) [4] and SCORing AD (SCORAD) [5] scores are the most common clinical measurement tools used to assess AD severity, with greater preference for EASI [6]. However, the EASI encounters limitations owing to its moderate interrater reliability and the lack of interpretability data, particularly in defining the severity ranges; mild, moderate, and severe AD [7, 8]. Additionally, the EASI assigns equal weight to extent and severity, potentially leading to a heterogeneous patient population with the same EASI score [9]. Thus, there is a growing interest in exploring accurate and objective approaches for evaluating the severity of skin diseases.

Recently, corneocyte surface topography has been suggested as a potential biomarker for evaluating skin diseases through the quantification of circular nano-size objects (CNOs) in the nanotexture of corneocytes [10–13]. CNOs are the nano-scale protrusions found on the corneocyte surface that have been associated with skin barrier impairment [14] and AD, although the exact nature and cause of CNOs remain unidentified [12]. This indicator enables non-invasive *ex vivo* analysis through stratum corneum (SC) tape stripping [15], which may serve as an objective and efficient tool for assessing AD severity.

However, the existing method, known as the Dermal Texture Index (DTI), identifies CNOs in corneocyte nanotexture images by relying on computer vision techniques that establish specific criteria, such as height, circularity index, and area of CNOs [10, 11]. Consequently, this approach is prone to inaccuracies due to the susceptibility of nano-imaging techniques to environmental noise. Moreover, DTI represents the CNO density calculated using the full range of corneocyte nanotexture images (20 x 20 μm^2), which may include ineffective areas with minimal CNO occurrence, such as ridges and structural occlusion on the corneocyte surface, potentially affecting the precision in density calculation.

In this study, we established an extensive database of corneocyte nanotexture images depicting various degrees of AD severity using our in-house high-speed dermal atomic force microscope (HS-DAFM) [16]. Subsequently, we leveraged the collected data to train state-of-the-art deep neural networks for accurately identifying corneocyte nanotexture features. To effectively address inaccuracies and artifacts during the nano-imaging process, this study analyzed the spatial distribution of the detected features, aiming to enhance robustness against susceptibility to environmental noise. For statistical analyses, this study focused on investigating corneocyte surface topography across various levels of AD severity based on their EASI scores, with the goal of advancing current methods used by physicians clinically to assess AD severity and providing a reliable and objective evaluation tool.

Material and Methods

Stratum corneum sample collection

This study included a total of 45 patients with AD and 15 healthy controls in Taiwan ($\geq$ 18 years). Ethics approval was obtained from the National Taiwan University Hospital (202204089RIND), and all participants gave written informed consent beforehand. The AD patients were divided into three severity groups based on their EASI scores: G1 (AD mild, EASI = 0.1-7.0), G2 (AD moderate, EASI = 7.1-21.0), and G3 (AD severe, EASI > 21.0), while the healthy controls were categorized as G4 (no AD history). We systematically collected SC samples from both lesional and non-lesional skin areas of each AD patient, ensuring a comprehensive representation of AD severity. No specific instructions were given for any interruption in topical treatment to ensure that the collected SC samples closely reflected real-world clinical scenarios. However, we acknowledge the potential variability in eczema severity at the lesional collection sites.

To obtain SC samples from the participants, we utilized a standardized tape-stripping procedure [17]. During the sampling process, we collected 5 consecutive circular adhesive tape strips (D101, 1.54 cm^2, D-Squame, Clinical & Derm, Dallas, TX, U.S.A.) from the volar side of the forearm, approximately 10 cm below the elbow crease. Each tape strip was pressed on the skin for 10 seconds using a pressure instrument (D500, D-Squame, Clinical & Derm, Dallas, TX, U.S.A.) to apply a constant pressure of 225 g/cm^2. Afterward, we gently removed each tape strip with tweezers and stored them individually in sampling vials.

The initial two strips were excluded from our analysis to mitigate any potential contamination or impurities on the skin surface. The third strip underwent RNA analysis [18], the fourth strip underwent surface topography imaging through our HS-DAFM, and the fifth strip underwent natural moisturizing factors (NMF) analysis [19]. The SC tapes designated for AFM topography measurement were stored at room temperature, whereas other tapes were stored immediately at -80 °C until further analysis. This study focuses on analyzing corneocyte surface topography as a potential biomarker for AD severity assessment. Results from RNA and NMF analyses will be detailed in upcoming publications.

Corneocyte surface topography dataset

For corneocyte topographic imaging, we employed a HS-DAFM using an aluminum-coated silicon-nitride AFM probe (spring constant of 0.03 N/m, CSC38/Al, MikroMasch, Germany) with a tip end radius of 8 nm. The SC samples were measured by the HS-DAFM in contact mode with constant height, resulting in corneocyte nanotexture images with a resolution of 512 x 512 pixels and an imaging area of 20 x 20 μm^2. To ensure consistent measurement quality, the contact force between the AFM tip and corneocyte surface was controlled to keep below 10 nN. For each SC sample, 10 random areas were selected to capture the surface topographical features of corneocytes, constructing a comprehensive dataset comprising over 1,000 corneocyte nanotexture images.

Image preprocessing

The nanotexture features of corneocytes are often challenging to discern due to the limited contrast level in AFM imaging [20] and their intricate structured backgrounds [21]. Therefore, we employed a variety of image processing techniques to enhance the

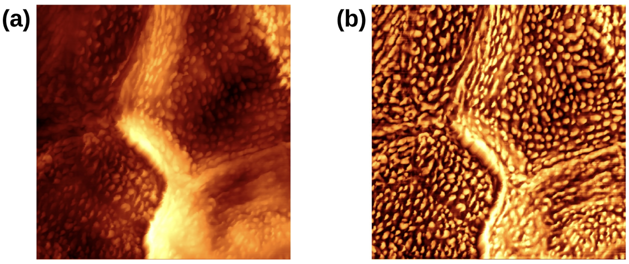

Figure 1. Demonstration of a corneocyte nanotexture image after applying image enhancement algorithms. (a) Original corneocyte nanotexture image captured by the HS-DAFM. (b) Processed image after applying image enhancement algorithms.

visibility of minute features (e.g., CNOs), while significantly suppressing the environmental noises. This enhancement facilitated subsequent image annotation and CNO identification.

First, we applied the mathematical morphology to extract shape and size information from the corneocyte nanotexture images by using structuring elements with various circular shapes and sizes [22, 23]. Subsequently, we employed the adaptive histogram equalization (AHE) technique to enhance local contrast, revealing finer morphological features of CNOs [24, 25]. Finally, the linear contrast stretching (LCS) technique was used to normalize the intensity levels by redistributing the intensity values across the entire intensity range [26]. Figure 1 shows the image enhancement outcome achieved by the proposed method for a corneocyte nanotexture image containing numerous CNOs sampled from an AD patient.

Training deep neural networks for CNO detection

The current state-of-the-art real-time object detectors are the You Only Look Once (YOLO) series [27–38], which have proven successful in various object detection applications due to their high detection accuracy and real-time inference speed [39–41]. Most YOLO object detectors use CSPNet [42] or ELAN [43] and their variants as the main computing units. For instance, the YOLOv8 [40, 41] model incorporates CSPDarknet53 [29] as its backbone feature extractor, while the latest YOLOv9 [38] model replaces ELAN with GELAN, employing CSPNet blocks with planned RepConv [37] as computational blocks. According to the evaluation results on the MS COCO dataset [44], YOLOv9 achieves a reduction of 49% in the number of parameters and 43% in computational workload compared to YOLOv8, while still demonstrating a 0.6% improvement in detection accuracy [38]. Another study validated the exceptional performance of YOLOv8 in detecting extremely small, pixelated objects that would be challenging for the human eye to identify [40].

To train YOLO object detectors for identifying and quantifying CNOs in corneocyte nanotexture images, we systematically selected a dataset of 300 corneocyte images with diverse AD severities. These images were meticulously labeled, contributing a comprehensive dataset with an average of over 350 annotated CNOs per image and a total of over 10,000 annotations across all images. Then, the dataset was randomly divided into three subsets for training and evaluating the object detection models: an 80% training set, a 10% validation set, and a 10% test set. In addition, we applied various data augmentation techniques, including adjustments to brightness, exposure, blur, and noise, to increase the size and diversity of the dataset. This augmentation process resulted in a threefold increase in the size of the training set [45].

In this study, we initially trained YOLOv8 and YOLOv9 models with default hyperparameters for 300 epochs. Subsequently, we compared the performance of YOLO models with varying complexities, namely YOLOv8-{N,S,M,L,X} and YOLOv9-{C,E}, to determine the optimal model for the CNO detection. Finally, a grid search with 100 epochs was conducted for each set of hyperparameters to identify the optimal configuration of our selected YOLO model. All models were initialized through transfer learning [46] with pre-trained weights from the MS COCO dataset and then underwent training on our custom dataset.

Spatial Analysis using kernel density estimation

Due to the high sensitivity of HS-DAFM to environmental noise and structural occlusion on the corneocyte, the calculation of CNO density is prone to variations. Additionally, certain regions on the corneocyte surface, characterized by ridges or fringes, exhibit minimal CNO occurrence, which influences the precision in calculating the density of CNOs.

To address these issues, we implemented an additional kernel density estimator (KDE), generating a density map that captured the spatial distribution of CNOs [47, 48]. The KDE's bandwidth (BW) selection, crucial for the density map's smoothness, was optimized through empirical method using the cross-validation [49]. Figure 2 illustrates the KDE density map with different BW selections. A smaller BW results in undersmoothing, while a larger BW leads to oversmoothing.

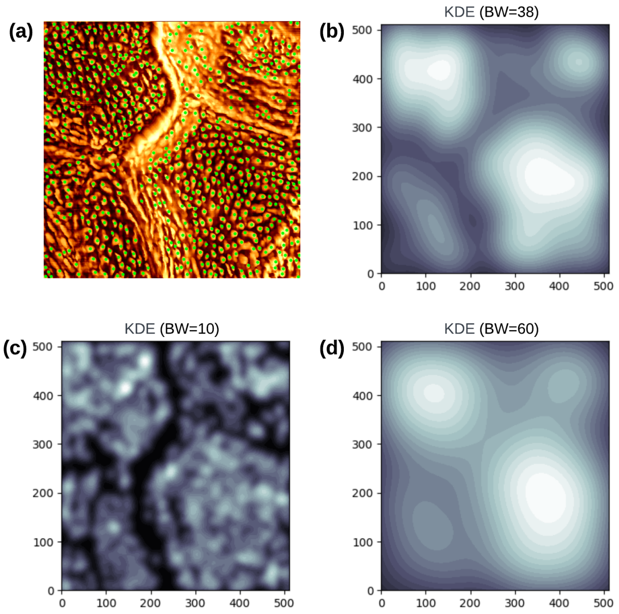

Figure 2. Determination of optimal BW for KDE via cross-validation. (a) Input corneocyte image with detected CNOs marked in green spots. (b) Selection of optimal bandwidth (BW=38). (c) Example of undersmoothing (BW=10). (d) Example of oversmoothing (BW=60).

Subsequently, a scaling of the KDE's density map into 25 layers provided detailed insights into CNO distribution, functioning as a practical tool to identify and exclude areas susceptible to occlusion or artifacts. For subsequent analysis, we utilized the average density of the central 5 layers from KDE's density map to calculate the CNO density on the corneocyte surface, ensuring a more reliable representation of the spatial distribution of CNOs. We termed the computed CNO density using KDE as the Effective Corneocyte Topographical Index (ECTI).

Analyses

Model evaluation and hyperparameters tuning

In this section, we conducted a comparative analysis of YOLO models with different architectures and sizes, evaluating their performance in terms of detection accuracy, inference speed, and model complexity. The average precision (AP) score [50, 51], a widely accepted metric for object detection models, was employed to evaluate the detection accuracy. AP provides a unified score by considering recall, precision, and intersection over union (IoU), thereby ensuring an unbiased assessment in performance. AP_{50} refers to the AP calculated using an IoU threshold of 0.5, while AP_{50-95} represents the average AP across different IoU thresholds, ranging from 0.5 to 0.95 in 0.05 increments [52]. Training and evaluation of all models were performed on an NVIDIA Tesla T4 GPU in Google Colab.

After conducting grid search for hyperparameter tuning, all models were trained with identical configurations to ensure comparable results. Each model underwent 300 epochs of training on our corneocyte dataset, utilizing an initial learning rate of 0.01, a finish learning rate of 0.0001, a momentum of 0.937, a weight decay of 0.005, a batch size of 4, and stochastic gradient descent (SGD) as

Table 1. Performance evaluation of YOLOv8 and YOLOv9 object detectors with different model complexities.

Model	#Parameter (M)	FLOPS (G)	Precision (%)	Recall (%)	AP_{50} (%)	AP_{50-95} (%)	Inference Speed (ms)
YOLOv8-N	3.0	8.1	79.4	78.5	86.5	48.4	34.8
YOLOv8-S	11.1	28.4	83.0	79.1	88.7	52.5	42.0
YOLOv8-M	25.8	78.7	84.9	83.5	90.8	57.6	50.3
YOLOv8-L	43.6	164.8	86.7	84.4	92.0	61.8	59.9
YOLOv8-X	68.1	257.4	87.4	84.4	92.2	63.4	64.1
YOLOv9-C	51.0	237.6	84.1	82.3	90.8	57.9	73.9
YOLOv9-E	69.3	243.3	84.6	83.8	91.4	58.7	85.2

[1] -{N, S, M, L, X} indicate nano, small, medium, large, and extra-large models; -{C, E} indicate compact and extended models.
[2] FLOPS indicates floating-point operations per second

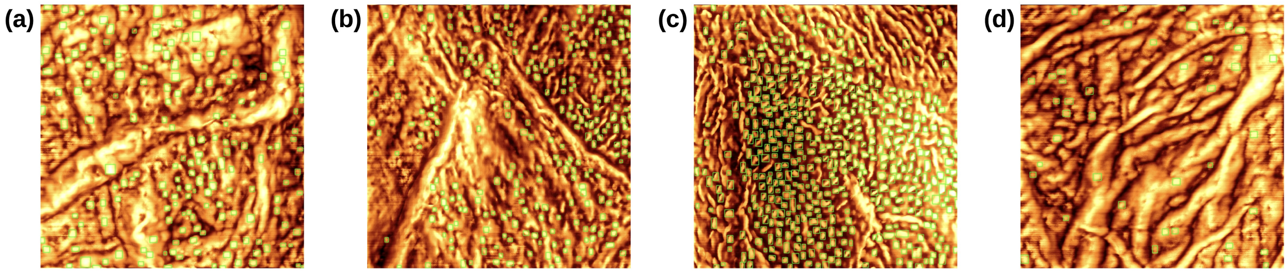

Figure 3. CNO quantification results using YOLOv8-X model with a confidence threshold of 0.3. (a) Mild AD sample (CNO count=180). (b)Moderate AD sample (CNO count=250). (c) Severe AD sample (CNO count=483). (d) Healthy control (CNO count=22).

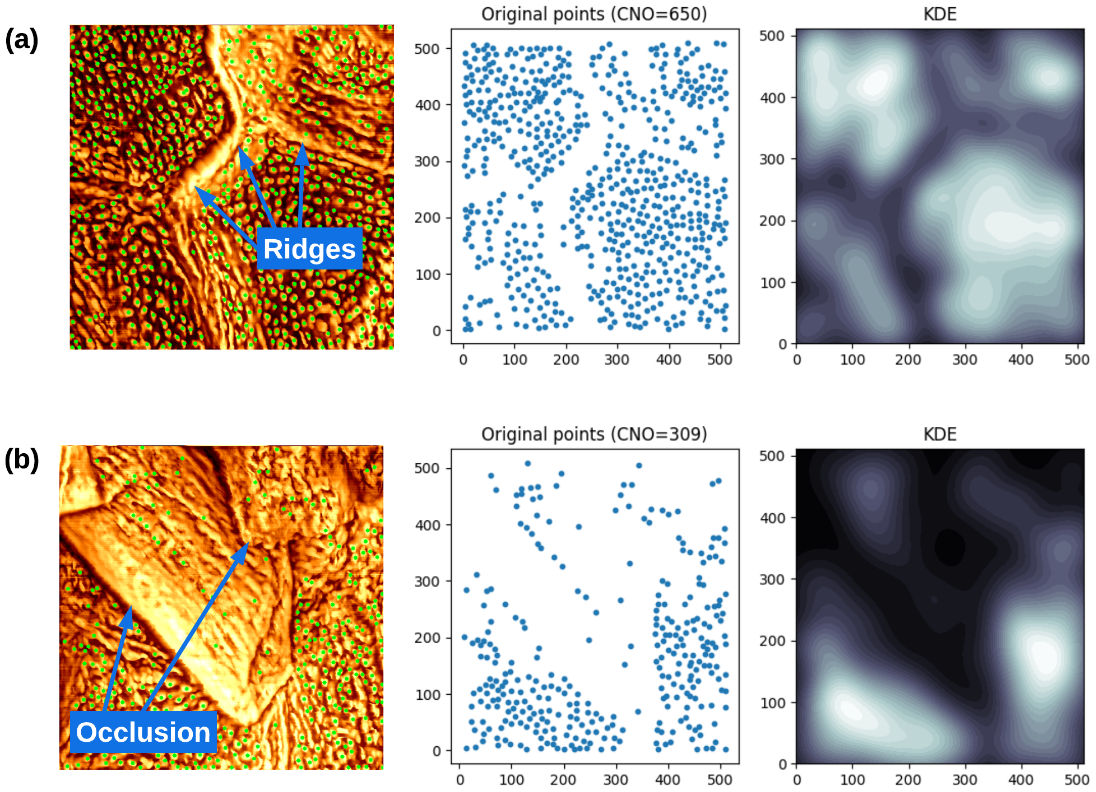

Figure 4. Spatial analysis of CNO distribution via KDE. (a) Visualization of corneocyte surface topography highlighting ridges. (b) Visualization of corneocyte surface topography showing occlusion.

the optimizer. Table 1 evaluates the performance of YOLOv8 and YOLOv9 models in terms of detection accuracy, model complexity, and inference speed. This evaluation was conducted using a test set comprising 30 annotated corneocyte nanotexture images, each with a resolution of 512 x 512 pixels.

The evaluation results revealed high accuracy performance in CNO detection across all YOLO models, surpassing 85% AP_{50} score consistently. Notably, increasing the model complexity positively influenced the accuracy of CNO detection, albeit resulting in a decrease in inference speed. The YOLOv8-X model demonstrated the highest detection accuracy, achieving an AP_{50} of 92.2% and an AP_{50-95} of 63.4%, thus making it the optimal choice for CNO detection.

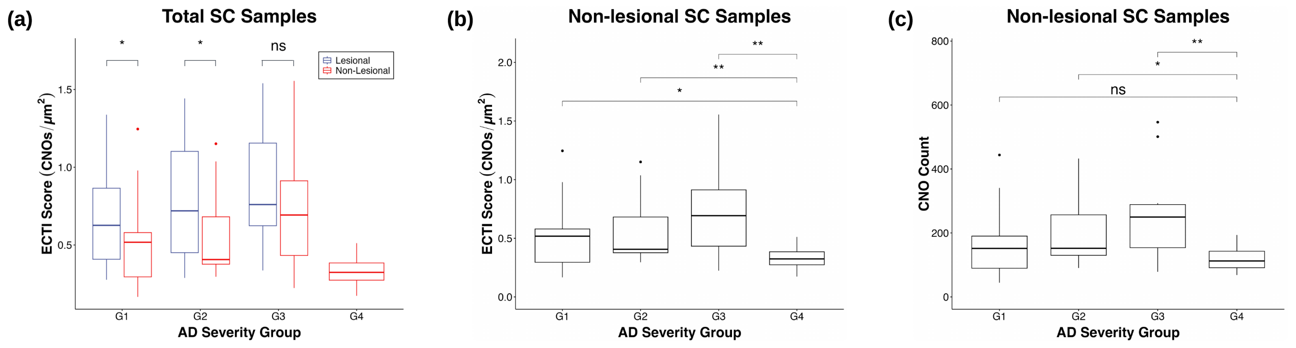

Figure 5. ECTI score and CNO count in the SC samples of AD patients (n = 15 in both lesional and non-lesional skin areas of each group) and healthy controls (n = 15). (a) Comparison of ECTI scores between lesional and non-lesional SC samples. (b) ECTI scores in the non-lesional SC samples using KDE. (c) CNO counts in the non-lesional SC samples without KDE application. Box plot notations: ns → not significant, $*p \leq 0.05$, $**p \leq 0.01$; AD severity groups according to EASI score: G1 → mild AD, G2 → moderate AD, G3 → severe AD, G4 → healthy controls.

Qualitative results

Figure 3 presents the qualitative results obtained by applying the YOLOv8-X object detector with a confidence threshold of 0.3 to the corneocyte nanotexture images from different AD severity groups. The results demonstrate the object detector's capability in quantifying CNOs even in the presence of vibrational noise introduced during topographic imaging.

Figure 4 illustrates the KDE process, where the algorithm generates a density map based on the spatial distribution of CNOs. This process effectively eliminates areas with ridges or occlusion, enhancing the precision in calculating CNO density.

Statistical analysis

The mean ECTI scores, obtained from the analysis of 10 nanotexture images per SC tape, were used for statistical analyses. Each AD group (G1, G2, G3) comprises 15 data points in both lesional and non-lesional SC samples, while the healthy control group (G4) comprises 15 data points in non-lesional SC samples only. Initially, samples in each AD severity group (G1, G2, G3, G4) underwent the Shapiro-Wilk normality test [53] respectively to assess data distribution. Given the non-normal distribution in most data groups, we adopted the Wilcoxon signed-rank test [54] to determine statistically significant difference between paired samples, specifically comparing the lesional and non-lesional SC samples collected from the same patient. Additionally, the Wilcoxon rank-sum test [55] was employed to identify significant differences between independent sample groups, namely the AD severity groups of G1, G2, G3, and G4. Samples with missing data or those that could not be paired for comparison were excluded from the analysis.

Figure 5(a) presents the statistical results via box plots, further dividing each AD severity group into lesional and non-lesional sampled areas. In general, the plot indicates a noticeable increase in ECTI scores corresponding to the severity of AD. A majority of AD severity groups exhibit significant differences between lesional and non-lesional SC samples, indicating a higher occurrence of CNOs in the lesional skin areas. Additionally, the healthy controls (G4) consistently demonstrate the lowest ECTI scores compared to other AD severity groups.

Figure 5(b) and (c) focus on the statistical analysis of non-lesional SC samples from each AD severity group (G1, G2, G3) in comparison to the healthy control group (G4). Figure 5(b) reveals significant differences between each AD severity group and the healthy controls, whereas Figure 5(c) fails to differentiate the mild AD group from the healthy controls by solely quantifying the CNOs across the full image range of 20 x 20 μm^2.

Discussion

In this study, we leveraged the state-of-the-art deep neural networks (YOLO) and spatial analysis techniques (KDE) to achieve precise identification of CNOs with high robustness against nano-imaging susceptibility. According to the evaluation results, our fine-tuned YOLOv8-X object detector demonstrated robust performance, achieving an overall accuracy of 92.2% in identifying CNOs. This emphasizes the effectiveness of our methodology in capturing subtle topographical features indicative of skin barrier impairment [14], despite the presence of environmental noise and structural occlusion on the corneocyte.

To examine the association between CNO density and AD severity, this study recruited 15 healthy controls and 45 patients with various AD severity based on their EASI scores. Each AD patient underwent a standardized tape-stripping procedure to collect SC samples from both lesional and non-lesional skin areas on the volar side of the forearm. Due to the stochastic distribution of eczema severity across sampled skin sites, AD lesional samples may exhibit varying local lesion severity, thereby influencing the ECTI scores in statistical analysis. Previous studies revealed significant differences between healthy controls and AD skin samples without specifying the clinical scoring of AD severity [12], thus lacking in-depth analysis in AD severity assessment.

Statistical analyses revealed significant differences in ECTI scores between SC samples of varying AD severity and healthy controls, in both AD lesional and non-lesional skin areas, with higher density observed in more severe cases of AD. Additionally, a comparative analysis was conducted to calculate the CNO density without applying spatial analysis techniques (KDE), reflecting the methodology of the existing biomarker (DTI) for skin disorders. DTI calculates the CNO density using the full range of corneocyte nanotexture images without excluding ineffective areas such as ridges and occlusions. Thus, the statistical result revealed a higher variance in the CNO density calculation, resulting in an inability to distinguish the mild AD severity group from healthy controls.

The integration of our findings into clinical practice has the potential to advance AD severity assessment, providing a non-invasive, objective, and precise evaluation method. Clinicians could employ corneocyte nanotexture analysis as a simple and efficient evaluation tool to monitor disease progression, assess treatment efficacy, and personalize therapeutic interventions for routine clinical use. Moreover, our study paves the way for further research into understanding the pathophysiology of AD and exploring novel therapeutic targets.

In future work, we aim to establish a comprehensive database comprising corneocyte nanotexture images that represent a broad spectrum of skin diseases and conditions. This expansion will enhance the versatility of our analytical approaches, enabling the di-

agnosis of a wide range of skin diseases. Moreover, we will leverage the corneocyte nanotexture database to refine our deep learning models and image processing algorithms, ensuring their adaptability for various applications. Our ongoing efforts will focus on extracting valuable insights from corneocyte nanotexture features, thereby providing a reliable and interpretable framework for evaluating skin health through corneocyte nanotexture analysis.

Conclusion

This article introduced a novel methodology for the accurate identification and density estimation of CNOs within the corneocyte surface topography, suggesting its potential as a biomarker for assessing AD severity. The ECTI score, obtained by analyzing the spatial distribution of CNOs using KDE, demonstrated notable robustness against nano-imaging susceptibility and revealed significant differences between AD skin samples of varying severity and healthy controls, in both lesional and non-lesional skin areas. In future work, we will expand our database to include various skin diseases and refine our models for broader applications, aiming to provide a reliable framework for evaluating skin health through corneocyte nanotexture analysis.

Availability of Source Code and Requirements

- Project name: Deep Learning-Based Skin Nanotexture Analysis using Atomic Force Microscope
- Project home page: https://gitlab.gbar.dtu.dk/jenhw/leo_skin_analysis
- Operating system(s): Platform independent
- Programming language: Python 3.11.4
- Other requirements: Python 3.10+, matplotlib 3.7.2, numpy 1.25.1, opencv-python 4.8.0.74, scipy 1.11.1, scikit-image 0.21.0, scikit-learn 1.3.1, ultralytics 8.0.132, customtkinter 5.2.1
- License: PSF, BSD, Apache, AGPL-3.0

Data Availability

The data supporting the findings of this study are available from the corresponding author upon request.

Abbreviations

AD: atopic dermatitis; EASI: Eczema Area and Severity Index; SCORAD: SCORing AD; CNO: circular nano-size object; SC: stratum corneum; DTI: Dermal Texture Index; HS-DAFM: high-speed dermal atomic force microscope; NMF: natural moisturizing factor; AHE: adaptive histogram equalization; LCS: linear contrast stretching; YOLO: You Only Look Once; KDE: kernel density estimation/estimator; BW: bandwidth; ECTI: Effective Corneocyte Topographical Index; AP: average precision; IoU: intersection over union; SGD: stochastic gradient descent; FLOPS: floating-point operations per second.

Competing Interests

The authors declare that they have no competing interests.

Funding

This project has received funding from the LEO Foundation under the open competition grant agreement No. LF-OC-20-000370; the Novo Nordisk Foundation under the Pioneer Innovator grant agreement No. NNF22OC0076607; the National Science and Technology Council of Taiwan (NSTC 112-2314-B-002-074-MY3); and the Intelligent Drug Delivery and Sensing using Microcontainers and Nanomechanics (IDUN).

Authors' Contributions

J.-H.W. developed the source code, annotated the dataset, and trained deep networks with support from J.P.; C.-W.D. designed the protocols for and carried out the collection of the dataset; I.J., M.O.C., and S.K. provided technical feedback and interpretation of all results throughout the development phase; S.S. provided feedback on the biological application of the tool; C.-Y.C. and E.E.-T.H. contributed to the critical revision of the manuscript; C.-Y.C., J.P.T., and E.E.-T.H. designed the project. All authors contributed to and approved the final manuscript.

References

1. Langan SM, Irvine AD, Weidinger S. Atopic dermatitis. The Lancet 2020;396(10247):345–360.
2. Barbarot S, Auziere S, Gadkari A, Girolomoni G, Puig L, Simpson EL, et al. Epidemiology of atopic dermatitis in adults: Results from an international survey. Allergy 2018 6;73:1284–1293.
3. Drucker AM, Wang AR, Li WQ, Sevetson E, Block JK, Qureshi AA. The Burden of Atopic Dermatitis: Summary of a Report for the National Eczema Association. Journal of Investigative Dermatology 2017;137(1):26–30.
4. Hanifin JM, Thurston M, Omoto M, Cherill R, Tofte SJ, Graeber M, et al. The eczema area and severity index (EASI): assessment of reliability in atopic dermatitis. Experimental Dermatology 2001 2;10:11–18.
5. Kunz B, Oranje AP, Labrèze L, Stalder JF, Ring J, Taïeb A. Clinical Validation and Guidelines for the SCORAD Index: Consensus Report of the European Task Force on Atopic Dermatitis. Dermatology 1997;195:10–19.
6. Zhao CY, Tran AQT, Lazo-Dizon JP, Kim J, Daniel BS, Venugopal SS, et al. A pilot comparison study of four clinician-rated atopic dermatitis severity scales. British Journal of Dermatology 2015 8;173:488–497.
7. Schmitt J, Langan S, Deckert S, Svensson A, von Kobyletzki L, Thomas K, et al. Assessment of clinical signs of atopic dermatitis: A systematic review and recommendation. Journal of Allergy and Clinical Immunology 2013 12;132:1337–1347.
8. Thomas KS. EASI does it: a comparison of four eczema severity scales. British Journal of Dermatology 2015 8;173:316–317.
9. Hanifin JM, Baghoomian W, Grinich E, Leshem YA, Jacobson M, Simpson EL. The Eczema Area and Severity Index—A Practical Guide. Dermatitis 2022 5;33:187–192.
10. Riethmuller C, McAleer MA, Koppes SA, Abdayem R, Franz J, Haftek M, et al. Filaggrin breakdown products determine corneocyte conformation in patients with atopic dermatitis. Journal of Allergy and Clinical Immunology 2015 12;136:1573–1580.e2.
11. Franz J, Beutel M, Gevers K, Kramer A, Thyssen JP, Kezic S, et al. Nanoscale alterations of corneocytes indicate skin disease. Skin Research and Technology 2016 5;22:174–180.
12. Engebretsen KA, Bandier J, Kezic S, Riethmüller C, Heegaard NHH, Carlsen BC, et al. Concentration of filaggrin monomers, its metabolites and corneocyte surface texture in individuals with a history of atopic dermatitis and controls. Journal of the European Academy of Dermatology and Venereology 2018 5;32:796–804.
13. Riethmüller C. Assessing the skin barrier via corneocyte morphometry. Experimental Dermatology 2018 8;27:923–930.

14. de Boer FL, van der Molen HF, Kezic S. Epidermal biomarkers of the skin barrier in atopic and contact dermatitis. Contact Dermatitis 2023 10;89:221–229.
15. Lademann J, Jacobi U, Surber C, Weigmann HJ, Fluhr JW. The tape stripping procedure – evaluation of some critical parameters. European Journal of Pharmaceutics and Biopharmaceutics 2009 6;72:317–323.
16. Liao HS, Akhtar I, Werner C, Slipets R, Pereda J, Wang JH, et al. Open-source controller for low-cost and high-speed atomic force microscopy imaging of skin corneocyte nanotextures. HardwareX 2022 10;12:e00341.
17. Dapic I, Jakasa I, Yau NLH, Kezic S, Kammeyer A. Evaluation of an HPLC Method for the Determination of Natural Moisturizing Factors in the Human Stratum Corneum. Analytical Letters 2013 9;46:2133–2144.
18. Inoue T, Kuwano T, Uehara Y, Yano M, Oya N, Takada N, et al. Non-invasive human skin transcriptome analysis using mRNA in skin surface lipids. Communications Biology 2022 3;5:215.
19. Kezic S, Kammeyer A, Calkoen F, Fluhr JW, Bos JD. Natural moisturizing factor components in the stratum corneum as biomarkers of filaggrin genotype: evaluation of minimally invasive methods. British Journal of Dermatology 2009 11;161:1098–1104.
20. Kienberger F, Pastushenko VP, Kada G, Puntheeranurak T, Chtcheglova L, Riethmueller C, et al. Improving the contrast of topographical AFM images by a simple averaging filter. Ultramicroscopy 2006 6;106:822–828.
21. Kimori Y. Mathematical morphology-based approach to the enhancement of morphological features in medical images. Journal of Clinical Bioinformatics 2011;1:33.
22. Haralick RM, Sternberg SR, Zhuang X. Image Analysis Using Mathematical Morphology. IEEE Transactions on Pattern Analysis and Machine Intelligence 1987 7;PAMI-9:532–550.
23. Oh J, Hwang H. Feature enhancement of medical images using morphology-based homomorphic filter and differential evolution algorithm. International Journal of Control, Automation and Systems 2010 8;8:857–861.
24. Zhu H, Chan FHY, Lam FK. Image Contrast Enhancement by Constrained Local Histogram Equalization. Computer Vision and Image Understanding 1999 2;73:281–290.
25. Zimmerman JB, Cousins SB, Hartzell KM, Frisse ME, Kahn MG. A psychophysical comparison of two methods for adaptive histogram equalization. Journal of Digital Imaging 1989 5;2:82–91.
26. Abdul-Nasir AS, Mashor MY, Mohamed Z. Modified Global and Modified Linear Contrast Stretching Algorithms: New Colour Contrast Enhancement Techniques for Microscopic Analysis of Malaria Slide Images. Computational and Mathematical Methods in Medicine 2012;2012:1–16.
27. Redmon J, Divvala S, Girshick R, Farhadi A. You Only Look Once: Unified, Real-Time Object Detection. In: 2016 IEEE Conference on Computer Vision and Pattern Recognition (CVPR); 2016. p. 779–788.
28. Redmon J, Farhadi A. YOLO9000: Better, Faster, Stronger. arXiv preprint arXiv:161208242 2016;.
29. Redmon J, Farhadi A. YOLOv3: An Incremental Improvement. arXiv preprint arXiv:180402767 2018;.
30. Bochkovskiy A, Wang CY, Liao HYM. YOLOv4: Optimal Speed and Accuracy of Object Detection. arXiv preprint arXiv:200410934 2020;.
31. Ge Z, Liu S, Wang F, Li Z, Sun J. YOLOX: Exceeding YOLO Series in 2021. arXiv preprint arXiv:210708430 2021;.
32. Wang CY, Bochkovskiy A, Liao HYM. Scaled-YOLOv4: Scaling Cross Stage Partial Network. arXiv preprint arXiv:201108036 2021;.
33. Chen Y, Yuan X, Wu R, Wang J, Hou Q, Cheng MM. YOLO-MS: Rethinking Multi-Scale Representation Learning for Real-time Object Detection. arXiv preprint arXiv:230805480 2023;.
34. Huang L, Li W, Shen L, Fu H, Xiao X, Xiao S. YOLOCS: Object Detection based on Dense Channel Compression for Feature Spatial Solidification. arXiv preprint arXiv:230504170 2023;.
35. Li C, Li L, Geng Y, Jiang H, Cheng M, Zhang B, et al. YOLOv6 v3.0: A Full-Scale Reloading. arXiv preprint arXiv:230105586 2023;.
36. Wang C, He W, Nie Y, Guo J, Liu C, Han K, et al. Gold-YOLO: Efficient Object Detector via Gather-and-Distribute Mechanism. arXiv preprint arXiv:230911331 2023;.
37. Wang CY, Bochkovskiy A, Liao HYM. YOLOv7: Trainable Bag-of-Freebies Sets New State-of-the-Art for Real-Time Object Detectors. In: 2023 IEEE/CVF Conference on Computer Vision and Pattern Recognition (CVPR); 2023. p. 7464–7475.
38. Wang CY, Yeh IH, Liao HYM. YOLOv9: Learning What You Want to Learn Using Programmable Gradient Information. arXiv preprint arXiv:240213616 2024;.
39. Li C, Li L, Jiang H, Weng K, Geng Y, Li L, et al. YOLOv6: A Single-Stage Object Detection Framework for Industrial Applications. arXiv preprint arXiv:220902976 2022;.
40. Rahman S, Rony JH, Uddin J, Samad MA. Real-Time Obstacle Detection with YOLOv8 in a WSN Using UAV Aerial Photography. Journal of Imaging 2023;9(10).
41. Khare OM, Gandhi S, Rahalkar AM, Mane S. YOLOv8-Based Visual Detection of Road Hazards: Potholes, Sewer Covers, and Manholes. arXiv preprint arXiv:231100073 2023 10;.
42. Wang C, Liao HM, Wu Y, Chen P, Hsieh J, Yeh I. CSPNet: A New Backbone that can Enhance Learning Capability of CNN. In: 2020 IEEE/CVF Conference on Computer Vision and Pattern Recognition Workshops (CVPRW) Los Alamitos, CA, USA: IEEE Computer Society; 2020. p. 1571–1580.
43. Wang CY, Liao HYM, Yeh IH. Designing Network Design Strategies Through Gradient Path Analysis. arXiv preprint arXiv:221104800 2022;.
44. Lin TY, Maire M, Belongie S, Hays J, Perona P, Ramanan D, et al. In: Microsoft COCO: Common Objects in Context Springer International Publishing; 2014. p. 740–755.
45. Shorten C, Khoshgoftaar T. A survey on Image Data Augmentation for Deep Learning. Journal of Big Data 2019 07;6.
46. Xu X, Zhang H, Ma Y, Liu K, Bao H, Qian X. TranSDet: Toward Effective Transfer Learning for Small-Object Detection. Remote Sensing 2023 7;15:3525.
47. Chen YC. A tutorial on kernel density estimation and recent advances. Biostatistics & Epidemiology 2017;1(1):161–187.
48. Węglarczyk S. Kernel density estimation and its application. ITM Web of Conferences 2018 11;23:00037.
49. Heidenreich NB, Schindler A, Sperlich S. Bandwidth selection for kernel density estimation: a review of fully automatic selectors. AStA Advances in Statistical Analysis 2013 10;97:403–433.
50. Everingham M, Eslami SMA, Gool LV, Williams CKI, Winn J, Zisserman A. The Pascal Visual Object Classes Challenge: A Retrospective. International Journal of Computer Vision 2015 1;111:98–136.
51. Russakovsky O, Deng J, Su H, Krause J, Satheesh S, Ma S, et al. ImageNet Large Scale Visual Recognition Challenge. International Journal of Computer Vision 2015 12;115:211–252.
52. Everingham M, Gool LV, Williams CKI, Winn J, Zisserman A. The Pascal Visual Object Classes (VOC) Challenge. International Journal of Computer Vision 2010 6;88:303–338.
53. Shapiro SS, Wilk MB. An Analysis of Variance Test for Normality (Complete Samples). Biometrika 1965 12;52:591.
54. Rey D, Neuhäuser M. In: Wilcoxon-Signed-Rank Test Springer Berlin Heidelberg; 2011. p. 1658–1659.
55. Fay MP, Proschan MA. Wilcoxon-Mann-Whitney or t-test? On assumptions for hypothesis tests and multiple interpretations of decision rules. Statistics Surveys 2010 1;4.
