# Supplementary material for: Stratum corneum nanotexture feature detection using deep learning and spatial analysis: a noninvasive tool for skin barrier assessment
Source: Gigascience. 2024 Dec 4;13:giae095. doi: 10.1093/gigascience/giae095 (PMC11629979; doi:10.1093/gigascience/giae095)
Supplement: giae095_GIGA-D-24-00100_Revision_1 [file giae095_giga-d-24-00100_revision_1.pdf]

GigaScience

Stratum corneum nanotexture feature detection using deep learning and spatial analysis: a non-invasive tool for skin barrier assessment

--Manuscript Draft--

Manuscript Number:	GIGA-D-24-00100R1
Full Title:	Stratum corneum nanotexture feature detection using deep learning and spatial analysis: a non-invasive tool for skin barrier assessment
Article Type:	Research
Funding Information:	LEO Fondet (LF-OC-20-000370) — Prof. Edwin En-Te Hwu<br>Novo Nordisk Fonden (NNF22OC0076607) — Prof. Edwin En-Te Hwu<br>National Science and Technology Council (NSTC 112-2314-B-002-074-MY3) — Prof. Chia-Yu Chu
Abstract:	Background: Corneocyte surface nanoscale topography (nanotexture) has recently emerged as a potential biomarker for inflammatory skin diseases, such as atopic dermatitis (AD). This assessment method involves quantifying circular nano-size objects (CNOs) in corneocyte nanotexture images, enabling non-invasive analysis via stratum corneum (SC) tape stripping. Current approaches for identifying CNOs rely on computer vision techniques with specific geometric criteria, resulting in inaccuracies due to the susceptibility of nano-imaging techniques to environmental noise and structural occlusion on the corneocyte.<br>Results: This study recruited 45 AD patients and 15 healthy controls, evenly divided into four severity groups based on their Eczema Area and Severity Index (EASI) scores. Subsequently, we collected a dataset of over 1,000 corneocyte nanotexture images using our in-house high-speed dermal atomic force microscope. This dataset was utilized to train state-of-the-art deep learning object detectors for identifying CNOs. Additionally, we implemented a kernel density estimator (KDE) to analyze the spatial distribution of CNOs, excluding ineffective regions with minimal CNO occurrence, such as ridges and occlusions, thereby enhancing accuracy in density calculations. After fine-tuning, our detection model achieved an overall accuracy of 91.4% in detecting CNOs.<br>Conclusions: By integrating deep learning object detector with spatial analysis algorithms, we developed a precise methodology for calculating CNO density, termed the Effective Corneocyte Topographical Index (ECTI). The ECTI demonstrated exceptional robustness to nano-imaging artifacts and presents substantial potential for advancing AD diagnostics by effectively distinguishing between SC samples of varying AD severity and healthy controls.
Corresponding Author:	Edwin En-Te Hwu<br>Technical University of Denmark: Danmarks Tekniske Universitet<br>Kongens Lyngby, DENMARK
Corresponding Author Secondary Information:	
Corresponding Author's Institution:	Technical University of Denmark: Danmarks Tekniske Universitet
Corresponding Author's Secondary Institution:	
First Author:	Jen-Hung Wang
First Author Secondary Information:	
Order of Authors:	Jen-Hung Wang<br>Jorge Pereda<br>Ching-Wen Du<br>Chia-Yu Chu, ORCID: 0000-0002-9370-3279

Powered by Editorial Manager® and ProduXion Manager® from Aries Systems Corporation

	Maria Oberländer Christensen
	Sanja Kezic
	Ivone Jakasa
	Jacob P. Thyssen
	Sreeja Satheesh
	Edwin En-Te Hwu
**Order of Authors Secondary Information:**	
**Response to Reviewers:**	Reviewer #1:<br>1. The paper utilizes YOLO object detection methods, including the recently proposed YOLOv8 and YOLOv9. Table 1 demonstrates that achieving optimal performance across all metrics is not feasible with a singular approach. Could you elaborate on the reasons behind this phenomenon?<br><br>Response:<br>We thank the reviewer's comment. The average precision (AP) score is a widely recognized metric for evaluating object detection models [1,2], as it provides a comprehensive assessment by combining recall, precision, and intersection over union (IoU) into a single measure. However, precision and recall often exhibit an inverse relationship, where improving one typically compromises the other. Consequently, achieving optimal performance across all metrics with a single approach is not feasible due to these inherent trade-offs.<br>Since precision and recall values are typically omitted in evaluations of object detection models [3,4], we focused on AP50 and AP50-95 scores [5] in the revised manuscript. This decision provides a clearer and more concise representation of our models' performance, enabling readers to more accurately assess the results.<br>Modified section in the manuscript: Analyses - Comparative analysis of deep learning object detector<br><br>2. Have you explored other object detection methods, such as two-stage methods like Mask R-CNN and one-stage methods like SSD, in addition to YOLO?<br><br>Response:<br>We appreciate the reviewer's suggestion. To offer a more comprehensive comparison for selecting object detection models in identifying circular nano-size objects (CNOs), we also trained the recently proposed end-to-end Transformer-based detectors (RT-DETR) [4,6] alongside the latest YOLOv10 models [3]. Following the reviewer's suggestion, we compare the performance of these state-of-the-art object detectors, allowing for a more informed decision in selecting the most effective model for our specific application.<br>Modified section in the manuscript: Material and Methods - Training deep learning object detectors for CNO detection<br><br>3. For experimental analysis, it is better to present quantitative visualization results of comparative methods, which can be shown in a figure.<br><br>Response:<br>We have now included an ablation study on kernel density estimation (KDE). This analysis quantifies the effect of KDE on the variability of CNO density calculations across AD severity groups, demonstrating the robustness of our approach.<br>Modified section in the manuscript: Analyses - Ablation study on KDE<br><br>4. Why did the authors choose a confidence threshold of 0.3? Have they experimented with other threshold values?<br><br>Response:<br>We thank the reviewer's suggestion. In the revised manuscript, we have updated the method for selecting the confidence threshold. We now choose the confidence threshold based on the F1-confidence curve [7] to ensure optimal balance between precision and recall. This approach allows us to determine the threshold that maximizes the F1 score, providing a more reliable criterion for evaluating model

Powered by Editorial Manager® and ProduXion Manager® from Aries Systems Corporation

performance.
Modified section in the manuscript: Analyses - Qualitative results

5. Recently, YOLOv10 has been proposed as an improvement over YOLOv9 (https://github.com/THU-MIG/yolov10). The authors might consider evaluating detection performance using YOLOv10.

Response:
We followed the reviewer's comment then trained and evaluated the latest YOLOv10 models for CNO detection, comparing their performance with Transformer-based models (RT-DETRv2) [4,6] in the revised manuscript.
Modified section in the manuscript: Analyses - Comparative analysis of deep learning object detectors

Reviewer #2:
1. What is the theoretical support for the estimation of sample size?

Response:
The sample size was estimated using Cochran's formula, based on a 6.7% prevalence of AD in the Taiwanese population [8]. The estimation indicated that at least 41 participants would be required to achieve an 80% confidence level, ensuring the true value falls within ±5% of the measured value [9,10]. In our study, we recruited a total of 45 AD patients and 15 healthy controls (≥18 years) in Taiwan, satisfying the calculated requirements.
The selection of the 20x20 μm^2 scanning range is based on the article "Nanoscale alterations of corneocytes indicate skin disease" (https://doi.org/10.1111/srt.12247), which details the typical dimensions of CNO (273 nm height and 305 nm width). This ensures that the chosen scanning range is appropriate for capturing the relevant nanoscale features.
Additionally, to calibrate the high-speed dermal atomic force microscope (HS-DAFM) scanner, a piece of a data track layer (ca. 1x1 cm^2) from a rewritable DVD was used as the calibration sample [11]. The DVD data tracks are characterized by a fixed period of 740 nm and a defined depth of 160 nm, allowing precise scanner calibration by measuring them [12]. This ensured that the corneocyte nanotexture images were captured at a resolution of 512x512 pixels over an imaging area of 20x20 μm^2, providing consistency in the quality and scale of the measurements.
Modified section in the manuscript: Material and Methods - Stratum corneum sample collection / Corneocyte surface topography dataset

2. Please provide a more detailed introduction to the image preprocessing techniques and the selection of hyperparameters for the deep models. What are the considerations behind each choice? Please also provide the corresponding code for review.

Response:
The revised manuscript includes a more detailed explanation of the image preprocessing techniques, and the corresponding code is available in our GitHub repository under utils/img_preprocessing.py, with detailed comments for clarity. For hyperparameter selection, we followed the train-from-scratch settings outlined in [3,4] for both the YOLOv10 and RT-DETRv2 models, ensuring consistency with established protocols. Detailed hyperparameter settings for each model are provided in the Supplementary Table S1. and S2. for further reference.
Modified section in the manuscript: Material and Methods - Image preprocessing / Training deep learning object detectors for CNO detection

3. Please include additional comparisons with existing methods or biomarkers to better demonstrate the novelty and superiority of the proposed approach.

Response:
We appreciate the reviewer's suggestion. In the revised manuscript, we have included an ablation study comparing the CNO density calculated using the kernel density estimator (KDE) with the traditional method of calculating density over the entire area,

Powered by Editorial Manager® and ProduXion Manager® from Aries Systems Corporation

similar to the Dermal Texture Index (DTI) [13]. This analysis quantifies the effect of KDE on the variability of CNO density calculations across AD severity groups, demonstrating the robustness of our approach.
In addition, Figure 6(B) and (C) present statistical analyses of CNO density calculated with and without the application of KDE. Figure 6(B) shows that KDE more effectively distinguishes between mild AD and healthy controls in non-lesional SC samples, whereas the analysis without KDE fails to achieve this distinction, as shown in Figure 6(C).
Modified section in the manuscript: Analyses - Ablation study on KDE

4. The discussion on the potential limitations and sources of error in the study could be more extensive.

Response:
In response to the reviewer’s feedback, we have expanded the discussion on the potential limitations and sources of error in the revised manuscript. Key limitations include the variability in sample collection due to differences in local eczema severity, exact sampling locations, and individual skin conditions, which could introduce inconsistencies in the results. We also acknowledge that the generalizability of the Effective Corneocyte Topographical Index (ECTI) to other dermatological conditions beyond AD remains to be validated, necessitating further research.
Modified section in the manuscript: Discussion

References
[1]Everingham M, Eslami SMA, Van Gool L, Williams CKI, Winn J, Zisserman A. The Pascal Visual Object Classes Challenge: A Retrospective. International Journal of Computer Vision 2014;111(1):98–136. http://dx.doi.org/10.1007/s11263-014-0733-5.
[2]Russakovsky O, Deng J, Su H, Krause J, Satheesh S, Ma S, et al. ImageNet Large Scale Visual Recognition Challenge. International Journal of Computer Vision 2015;115(3):211–252. http://dx.doi.org/10.1007/s11263-015-0816-y.
[3]Wang A, Chen H, Liu L, Chen K, Lin Z, Han J, et al., YOLOv10: Real-Time End-to-End Object Detection; 2024. https://arxiv.org/abs/2405.14458.
[4]Zhao Y, LvW, Xu S,Wei J,Wang G, Dang Q, et al., DETRs Beat YOLOs on Real-time Object Detection; 2024. https://arxiv.org/abs/2304.08069.
[5]EveringhamM, Van Gool L,Williams CKI,Winn J, Zisserman A. The Pascal Visual Object Classes (VOC) Challenge. International Journal of Computer Vision 2009;88(2):303–338. http://dx.doi.org/10.1007/s11263-009-0275-4.
[6]LvW, Zhao Y, Chang Q, Huang K,Wang G, Liu Y, RT-DETRv2: Improved Baseline with Bag-of-Freebies for Real-Time Detection Transformer; 2024. https://arxiv.org/abs/2407.17140.
[7]Ganguly P, Methani NS, Khapra MM, Kumar P. A Systematic Evaluation of Object Detection Networks for Scientific Plots. Proceedings of the AAAI Conference on Artificial Intelligence 2021;35(2):1379–1387. http://dx.doi.org/10.1609/aaai.v35i2.16227.
[8]Chan TC, Wu NL,Wong LS, Cho YT, Yang CY, Yu Y, et al. Taiwanese Dermatological Association consensus for the management of atopic dermatitis: A 2020 update. Journal of the Formosan Medical Association 2021;120(1):429–442. http://dx.doi.org/10.1016/j.jfma.2020.06.008.
[9]Hajian-Tilaki K. Sample size estimation in diagnostic test studies of biomedical informatics. Journal of Biomedical Informatics 2014;48:193–204. http://dx.doi.org/10.1016/j.jbi.2014.02.013.
[10]Heinisch O. Cochran,W. G.: Sampling Techniques, 2. Aufl. John Wiley and Sons, New York, London 1963. Preis s. Biometrische Zeitschrift 1965;7(3):203–203. http://dx.doi.org/10.1002/bimj.19650070312.
[11]Liao HS, Akhtar I, Werner C, Slipets R, Pereda J, Wang JH, et al. Open-source controller for low-cost and high-speed atomic force microscopy imaging of skin corneocyte nanotextures. HardwareX 2022;12:e00341. https://doi.org/10.1016/j.ohx.2022.e00341.
[12]E.-T. Hwu, A. Boisen, Hacking CD/DVD/Blu-ray for biosensing. ACS Sens. 2018; 1222–1232, https://doi.org/10.1021/acssensors.8b00340.
[13]Riethmuller C,McAleerMA, Koppes SA, Abdayem R, Franz J, Haftek M, et al. Filaggrin breakdown products determine corneocyte conformation in patients with atopic dermatitis. Journal of Allergy and Clinical Immunology 2015;136:1573–1580.e2. https://doi.org/10.1016%2Fj.jaci.2015.04.042.

Powered by Editorial Manager® and ProduXion Manager® from Aries Systems Corporation

Additional Information:	
Question	Response
Are you submitting this manuscript to a special series or article collection?	No
**Experimental design and statistics**<br><br>Full details of the experimental design and statistical methods used should be given in the Methods section, as detailed in our Minimum Standards Reporting Checklist. Information essential to interpreting the data presented should be made available in the figure legends.<br><br>Have you included all the information requested in your manuscript?	Yes
**Resources**<br><br>A description of all resources used, including antibodies, cell lines, animals and software tools, with enough information to allow them to be uniquely identified, should be included in the Methods section. Authors are strongly encouraged to cite Research Resource Identifiers (RRIDs) for antibodies, model organisms and tools, where possible.<br><br>Have you included the information requested as detailed in our Minimum Standards Reporting Checklist?	Yes
**Availability of data and materials**<br><br>All datasets and code on which the conclusions of the paper rely must be either included in your submission or deposited in publicly available repositories (where available and ethically appropriate), referencing such data using a unique identifier in the references and in the “Availability of Data and Materials” section of your manuscript.	Yes

Have you have met the above requirement as detailed in our Minimum Standards Reporting Checklist?	

Powered by Editorial Manager® and ProduXion Manager® from Aries Systems Corporation

Manuscript

Click here to access/download;Manuscript;GIGA-D-24-00100_Revised Manuscript word document clean.docx

Stratum corneum nanotexture feature detection using deep learning and spatial analysis: a non-invasive tool for skin barrier assessment

Jen-HungWang[1], Jorge Pereda[1], Ching-Wen Du[1,2], Chia-Yu Chu[2,*],Maria Oberländer Christensen[3], Sanja Kezic[4], Ivone Jakasa[5], Jacob P. Thyssen[3], Sreeja Satheesh[6] and Edwin En-Te Hwu[1,*]

[1]Department of Health Technology, Technical University of Denmark, Denmark

[2]Department of Dermatology, National Taiwan University Hospital and National Taiwan University College of Medicine, Taipei, Taiwan

[3]Department of Dermatology, Bispebjerg and Frederiksberg Hospital (BFH). University Hospitals of Copenhagen, Copenhagen, Denmark

[4]Department of Public and Occupational Health, Amsterdam Public Health Research Institute, Amsterdam University Medical Center, Amsterdam, The Netherlands

[5]Laboratory for Analytical Chemistry, Department of Chemistry and Biochemistry, Faculty of Food Technology and Biotechnology, University of Zagreb, Zagreb, Croatia

[6]Institute of Solid State Physics, Leibniz University Hannover, Hannover, Germany

[*]Correspondence address:

Chia-Yu Chu, Department of Dermatology, National Taiwan University Hospital and National Taiwan University College of Medicine, Taipei, Taiwan. E-mail: chiayu@ntu.edu.tw;

Edwin En-Te Hwu, Department of Health Technology, Technical University of Denmark, Denmark. E-mail: etehw@dtu.dk

ORCID iDs:

Jen-Hung Wang [0000-0001-9214-5837]; Jorge Pereda [0000-0002-4802-0591]; Ching-Wen Du [0009-0006-5517-1845]; Chia-Yu Chu [0000-0002-9370-3279]; Maria Oberländer Christensen [0000-0001-7177-1054]; Sanja Kezic [0000-0002-1063-4547]; Ivone Jakasa [0000-0002-7961-4069]; Jacob P Thyssen [0000-0003-3770-1743]; Sreeja Satheesh [0009-0002-4239-2578]; Edwin En-Te Hwu [0000-0002-5971-4978];

Abstract

Background: Corneocyte surface nanoscale topography (nanotexture) has recently emerged as a potential biomarker for inflammatory skin diseases, such as atopic dermatitis (AD). This assessment method involves quantifying circular nano-size objects (CNOs) in corneocyte nanotexture images, enabling non-invasive analysis via stratum corneum (SC) tape stripping. Current approaches for identifying CNOs rely on computer vision techniques with specific geometric criteria, resulting in inaccuracies due to the susceptibility of nano-imaging techniques to environmental noise and structural occlusion on the corneocyte.

Results: This study recruited 45 AD patients and 15 healthy controls, evenly divided into four severity groups based on their Eczema Area and Severity Index (EASI) scores. Subsequently, we collected a dataset of over 1,000 corneocyte nanotexture images using our in-house high-speed dermal atomic force microscope. This dataset was utilized to train state-of-the-art deep learning object detectors for identifying CNOs. Additionally, we implemented a kernel density estimator (KDE) to analyze the spatial distribution of CNOs, excluding ineffective regions with minimal CNO occurrence, such as ridges and occlusions, thereby enhancing accuracy in density calculations. After fine-tuning, our detection model achieved an overall accuracy of 91.4% in detecting CNOs.

Conclusions: By integrating deep learning object detector with spatial analysis algorithms, we developed a precise methodology for calculating CNO density, termed the Effective Corneocyte Topographical Index (ECTI). The ECTI demonstrated exceptional robustness to nano-imaging artifacts and presents substantial potential for advancing AD diagnostics by effectively distinguishing between SC samples of varying AD severity and healthy controls.

Keywords: atopic dermatitis (AD), corneocyte surface topography, deep learning, object detection, kernel density estimator (KDE), atomic force microscope (AFM)

Introduction

Atopic dermatitis (AD) is a prevalent inflammatory skin disease, affecting approximately 20% of children and 5-10% of adults in high-income countries [1]. A multinational survey reported that 10-20% of adult AD patients experience severe symptoms [2]. The increasing severity of AD has been shown to significantly impact quality of life, yet reliable biomarkers for assessing disease severity are still lacking [3]. Therefore, finding an accurate measure is crucial for effective disease management and evaluating treatment efficacy.

The Eczema Area and Severity Index (EASI) [4] and SCORing AD (SCORAD) [5] scores are the commonly used clinical tools for assessing AD severity, with a preference for the EASI [6]. However, the EASI is limited by its moderate interrater reliability and a lack of interpretability data, particularly in defining the severity ranges of mild, moderate, and severe AD [7, 8]. Additionally, the EASI assigns equal weight to both extent and severity, potentially leading to a heterogeneous patient population with the same EASI score [9].

Recently, corneocyte surface nanoscale topography (nanotexture) has emerged as a potential biomarker for evaluating skin diseases, particularly through the quantification of circular nano-size objects (CNOs) in corneocyte nanotexture [10-13]. CNOs are nano-scale protrusions observed on the corneocyte surface that have been linked to skin barrier impairment [14] and AD, although their exact nature and underlying causes remain unidentified [12]. This biomarker enables non-invasive ex vivo analysis through stratum corneum (SC) tape stripping [15], which may serve as an objective and efficient tool for assessing AD severity.

However, the current method, known as the Dermal Texture Index (DTI), identifies CNOs in corneocyte nanotexture images by utilizing computer vision techniques that rely on specific criteria, such as height, circularity index, and area of CNOs [10, 11]. Consequently, this approach is prone to inaccuracies due to the susceptibility of nano-imaging techniques to environmental noise. Moreover, the DTI calculates CNO density across the entire corneocyte nanotexture image (20x20 μm^2), which may include ineffective regions with minimal CNO occurrence, such as ridges and structural occlusions on the corneocyte surface, potentially compromising the accuracy of density calculations.

In this study, we used our in-house high-speed dermal atomic force microscope (HS-DAFM) [16] to establish an extensive database of corneocyte nanotexture images, capturing various levels of AD severity. The collected data was then leveraged to train state-of-the-art deep learning object detectors for the accurate identification of corneocyte nanotexture features. To address potential inaccuracies and artifacts arising from the nano-imaging process, we further analyzed the spatial distribution of the detected features, aiming to enhance robustness in calculating CNO density. For statistical analyses, this study investigated variations in corneocyte surface topography across different levels of AD severity, as categorized by EASI scores. The objective was to improve current clinical methods used by physicians to assess AD severity, providing a more reliable and quantifiable evaluation tool.

Material and Methods

Stratum corneum sample collection

This study included a total of 45 AD patients and 15 healthy controls in Taiwan (≥ 18 years). Ethics approval was obtained from the National Taiwan University Hospital (202204089RIND), and all participants provided written informed consent prior to participation. The sample size was estimated using Cochran's formula, based on a 6.7% prevalence of AD in the Taiwanese population [17], with an 80% confidence level and a 5% margin of error [18, 19]. The AD patients were evenly divided into three severity groups of 15 patients each, based on their EASI scores: G1 (AD mild, EASI = 0.1-7.0), G2 (AD moderate, EASI = 7.1-21.0), and G3 (AD severe, EASI > 21.0). The healthy controls were categorized as G4 (no AD history). We systematically collected SC samples from both lesional and non-lesional skin areas of each AD patient, ensuring a comprehensive representation of AD severity. No specific instructions were given regarding the interruption of topical treatment, to ensure that the collected SC samples closely reflected real-world clinical scenarios. However, we acknowledge the potential influence of topical treatment at the lesional collection sites.

The SC samples were obtained using a standardized tape-stripping procedure [20]. During sampling, we collected 5 consecutive circular adhesive tape strips (D101, 1.54 cm^2, D-Squame, Clinical & Derm, Dallas, TX, U.S.A.) from the volar side of the forearm, approximately 10 cm below the elbow crease. Each tape strip was pressed onto the skin for 10 seconds using a pressure instrument (D500, D-Squame, Clinical & Derm, Dallas, TX, U.S.A.) to maintain a constant pressure of 225 g/cm^2. Subsequently, we gently removed each tape strip with tweezers and stored them individually in sampling vials.

The initial two strips were excluded from analysis to minimize potential contamination or impurities on the skin surface. The third strip underwent RNA analysis [21], the fourth strip was used for surface topography imaging with our HS-DAFM, and the fifth strip was analyzed for natural moisturizing factors (NMF) [22]. The SC tapes designated for AFM topography measurement were stored at room temperature, while the remaining tapes were immediately stored at -80 °C until further analysis. This study focused on analyzing corneocyte surface topography as a potential biomarker for AD severity assessment. Results from RNA and NMF analyses will be detailed in upcoming publications.

Corneocyte surface topography dataset

To measure corneocyte nanotexture, we utilized a HS-DAFM equipped with an aluminum-coated silicon-nitride AFM probe (spring constant of 0.03 N/m, CSC38/Al, MikroMasch, Germany) with a tip radius of 8 nm. The SC samples were measured in contact mode at a constant height, with the contact force maintained below 10 nN to ensure consistent measurement quality. The HS-DAFM scanner was calibrated using a piece of DVD data track layer (approximately 1x1 cm^2) as the calibration sample [23]. The DVD data tracks are characterized by a fixed period of 740 nm and a defined depth of 160 nm, allowing precise scanner calibration through their measurement.

For each SC sample, 10 random areas were selected to capture the surface topographical features of corneocytes, resulting in a comprehensive dataset of over 1,000 corneocyte nanotexture images. Each image was acquired at a resolution of 512x512 pixels, covering an imaging area of 20x20 μm^2. The scanning range was chosen based on findings from [11], which specify the typical dimensions of CNOs (273 nm in height and 305 nm in width), ensuring that the selected area is appropriate for capturing relevant nanoscale features.

Image preprocessing

Corneocyte nanotexture features are often challenging to discern due to the limited contrast level in AFM imaging [24] and their intricate structured backgrounds [25]. Therefore, we applied a series of image processing techniques to enhance the visibility of minute features, such as CNOs, while effectively suppressing environmental noise. This enhancement facilitated the subsequent process of image annotation and CNO detection.

Initially, we applied Gaussian filtering to smooth the raw images [26], followed by subtracting the mean intensity across each row to effectively mitigate striping artifacts in AFM imaging [27, 28]. Subsequently, the images were normalized to a range of 0.0 to 1.0 to ensure consistent intensity levels across all samples. Finally, disk-shaped morphological elements, with diameters of 9 and 15 pixels, were applied as percentile filters, systematically scanning the entire image to enhance local contrast and improve the visibility of subtle features, such as CNOs [29-31].

Figure 1 shows the result of the image enhancement algorithms, demonstrating improved visibility of CNOs in a corneocyte nanotexture image captured from an SC sample of an AD patient.

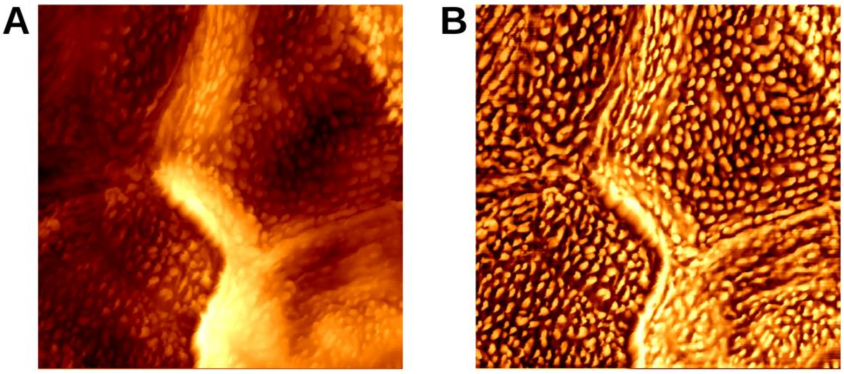

Figure 1. Demonstration of a corneocyte nanotexture image before and after applying the image enhancement algorithms. (A) Original corneocyte nanotexture image captured using HS-DAFM. (B) Enhanced image revealing clearer CNO contours.

Training deep learning object detectors for CNO detection

Object detection is a critical task in computer vision that involves identifying and localizing objects within an image, and it has become a widely used technology in fields ranging from autonomous driving [32, 33] to medical imaging [34, 35]. In this study, we evaluated the performance of two state-of-the-art deep learning object detection approaches — Convolutional Neural Network (CNN)-based detectors [36-40] and Transformer-based detectors [41-47] — specifically for identifying CNOs in corneocyte nanotexture images.

Among CNN-based models, the YOLO (You Only Look Once) series [38-40, 48-58] has emerged as the most popular framework for real-time object detection, renowned for its optimal balance between speed and accuracy [59-61]. The latest iteration, YOLOv10 [58], introduces notable advancements, such as non-maximum suppression (NMS)-free training and large-kernel convolutions, which enhance its efficiency and accuracy, particularly in the detection of small, intricate features [62, 63]. In contrast, Transformer-based detectors enable end-to-end object detection [64] by employing self-attention mechanisms, which eliminate the need for NMS post-processing. Building on this framework, RT-DETR (Real-Time Detection Transformer) [65, 66] further implements an efficient hybrid encoder and introduces uncertainty-minimal query selection to improve both accuracy and latency.

To train the object detectors, we systematically selected a dataset of 300 corneocyte nanotexture images with diverse AD severities. Each image was meticulously labeled, contributing a comprehensive dataset with an average of approximately 250 annotated CNOs per image and over 74,000 annotations in total. The dataset was then randomly split into three subsets for training and evaluating the object detectors: an 80% training set, a 10% validation set, and a 10% test set. Additionally, we applied a range of data augmentation techniques [67, 68] to expand the training set threefold, including adjustments to brightness (-25% to 25%), exposure (-15% to 15%), blur (up to 1 pixel), noise (up to 2% of pixels), and Mosaic augmentation [48].

In this study, we focused on fine-tuning YOLOv10 and RT-DETRv2 [66] models for CNO detection using our corneocyte nanotexture image dataset. Specifically, we compared the performance of various scales within each model, namely YOLOv10-{N, S, M, B, L, X} and RT-DETRv2-{S, M, L, X}, to determine the optimal configuration for CNO detection. All models were trained and evaluated on an NVIDIA Tesla T4 GPU in Google Colab, following the same train-from-scratch settings as in [58, 65], respectively. Due to computational limitations, we adjusted the batch size as necessary. Detailed hyperparameter settings for each model are provided in Supplementary Table S1. and S2. for further reference.

Spatial Analysis using kernel density estimator

The calculation of CNO density can exhibit significant variability due to the high sensitivity of nano-imaging techniques to environmental noise and structural occlusions on the corneocyte surface. Moreover, regions such as ridges or fringes on the corneocyte tend to have minimal CNO presence, which may compromise the accuracy of density calculations. This inherent variability in CNO distribution poses challenges in obtaining consistent and reliable density estimates.

To address these issues, we implemented a kernel density estimator (KDE) [69, 70] to generate a continuous, probabilistic density map that captures the spatial distribution of CNOs across the corneocyte surface. KDE provides a flexible framework to estimate densities from sparse and unevenly distributed data points, such as CNO coordinates, by smoothing the distribution over the entire surface. A critical parameter in KDE is the kernel's bandwidth (BW), which determines the smoothness of the density estimate. An overly small BW results in undersmoothing, amplifying minor variations and noise in the data, whereas an excessively large BW oversmooths the density map, potentially obscuring important structural details.

To optimize KDE performance, we empirically tuned the BW using cross-validation to balance between undersmoothing and oversmoothing [71]. This approach ensures that the density map accurately reflects the spatial variation in CNO distribution, while minimizing the influence of noise or occlusion artifacts. As shown in Figure 2, the selection of BW has a substantial impact on the KDE output, where smaller BW values emphasize localized variations while larger BW values result in a more homogenized density map. Additionally, we divided the KDE density map into 25 discrete layers to enable a more detailed analysis of CNO distribution across various regions of the corneocyte. This stratified method allowed us to isolate and exclude regions affected by occlusion or artifacts, thereby improving the robustness of the analysis.

For subsequent analyses, we calculated the CNO density on the corneocyte surface by averaging the density values from the central 5 layers of the KDE density map, ensuring a more reliable representation of CNO distribution. In this study, the CNO density calculated using KDE was termed the Effective Corneocyte Topographical Index (ECTI).

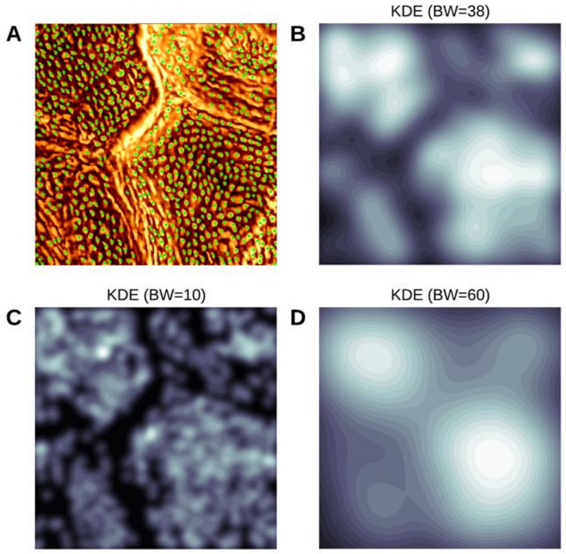

Figure 2. Optimal BW selection for KDE using cross-validation. (A) Corneocyte nanotexture image with detected CNOs marked as green spots. (B) Selected optimal BW=38. (C) Example of undersmoothing (BW=10). (D) Example of oversmoothing (BW=60).

Analyses

Comparative analysis of deep learning object detectors

In this section, we compare the performance of YOLOv10 and RT-DETRv2 models for CNO detection based on model scale, computational cost, detection accuracy, and inference speed. The standard average precision (AP) metrics [72, 73] were used to evaluate detection accuracy. AP provides a unified score by integrating metrics such as recall, precision, and intersection over union (IoU), ensuring an unbiased performance assessment. AP50 refers to the AP calculated at a fixed IoU threshold of 0.5, whereas AP50-95 represents the mean AP across uniformly sampled IoU thresholds from

0.50 to 0.95, with a step size of 0.05 [74]. The evaluation was conducted on a test set of 30 annotated corneocyte nanotexture images. In addition, latency was measured on an NVIDIA Tesla T4 GPU using TensorRT FP16 [75], with all test images resized to 512x512 pixels to align with the resolution of the corneocyte nanotexture images.

Table 1 presents the evaluation results of the YOLOv10 and RT-DETRv2 models, including the number of parameters, floating-point operations per second (FLOPs), AP at different IoU thresholds, and latency. Both object detectors achieve high AP50 scores above 83%; however, RT-DETRv2 exhibits lower AP50-95 scores compared to YOLOv10. The results show that YOLOv10 consistently outperforms RT-DETRv2 in detection accuracy across all model scales. Notably, the YOLOv10-L model achieves the highest accuracy, with an AP50 of 91.4% and an AP50-95 of 63.2%, exceeding the best-performing RT-DETRv2 variant (RT-DETRv2-S) with an AP50 of 87.6% and an AP50-95 of 39.6%.

In terms of inference speed, both models are capable of real-time object detection. However, when comparing models of similar scales, such as YOLOv10-B with RT-DETRv2-S and YOLOv10-X with RT-DETRv2-M, RT-DETRv2 generally demonstrates lower computational costs (FLOPs) and reduced latency.

Table 1. Performance comparison of YOLOv10 and RT-DETRv2 object detectors across various model scales. The table evaluates the models in terms of the number of parameters (M), FLOPS (G), AP50 (%), AP50-95 (%), and latency (ms).

Model	#Parameter (M)	FLOPS (G)	AP_{50} (%)	AP_{50-95} (%)	Latency (ms)
YOLOv10-N	2.7	8.2	89.6	51.4	3.33
YOLOv10-S	8.0	24.4	90.8	55.5	4.58
YOLOv10-M	16.5	63.4	91.3	59.7	7.17
YOLOv10-B	20.4	97.7	91.1	62.5	7.58
YOLOv10-L	25.7	126.3	91.4	63.2	9.01
YOLOv10-X	31.6	169.8	91.2	62.9	10.95
RT-DETRv2-S	20.0	60.0	87.6	39.6	5.51
RT-DETRv2-M	31.0	100.0	84.0	37.2	7.48
RT-DETRv2-L	42.0	136.0	84.3	33.4	13.50
RT-DETRv2-X	76.0	259.0	83.3	32.0	21.15

[1] -{N, S, M, B, L, X} indicate nano, small, medium, balanced, large, and extra-large models.

Qualitative results

Figure 3 presents the qualitative results of applying the fine-tuned YOLOv10-L model to detect CNOs on corneocyte nanotexture images with different AD severity levels (G1, G2, G3, G4). The confidence threshold was set to 0.141, as this value achieved the highest F1 score [76] of 0.85, providing an optimal balance between precision and recall. The F1-confidence curve for the fine-tuned YOLOv10-L model is provided in Supplementary Fig. S2. The results demonstrate the model's capability to accurately quantify in quantifying CNOs, even in the presence of vibrational noise introduced during topographic imaging.

Figure 4 presents the analysis results of CNO distribution using KDE, in which the algorithm generates a density map representing the spatial distribution of CNOs. This process effectively excludes regions with ridges or occlusions, thereby improving the accuracy of CNO density calculations.

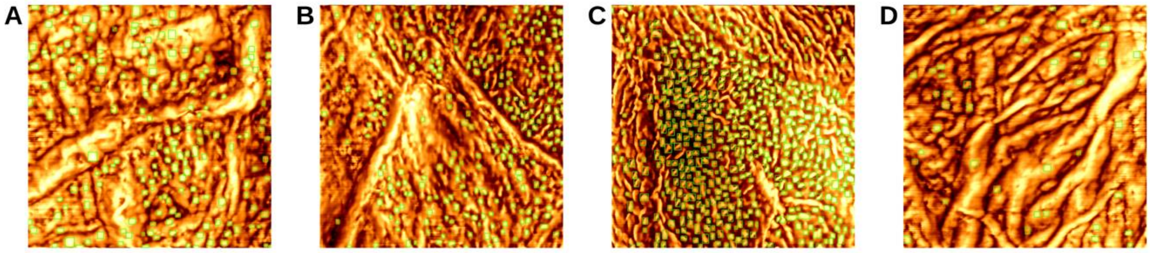

Figure 3. CNO detection results using YOLOv10-L model with a confidence threshold of 0.141. (A) Mild AD sample (CNO count=180). (B)Moderate AD sample (CNO count=250). (C) Severe AD sample (CNO count=483). (D) Healthy control (CNO count=22).

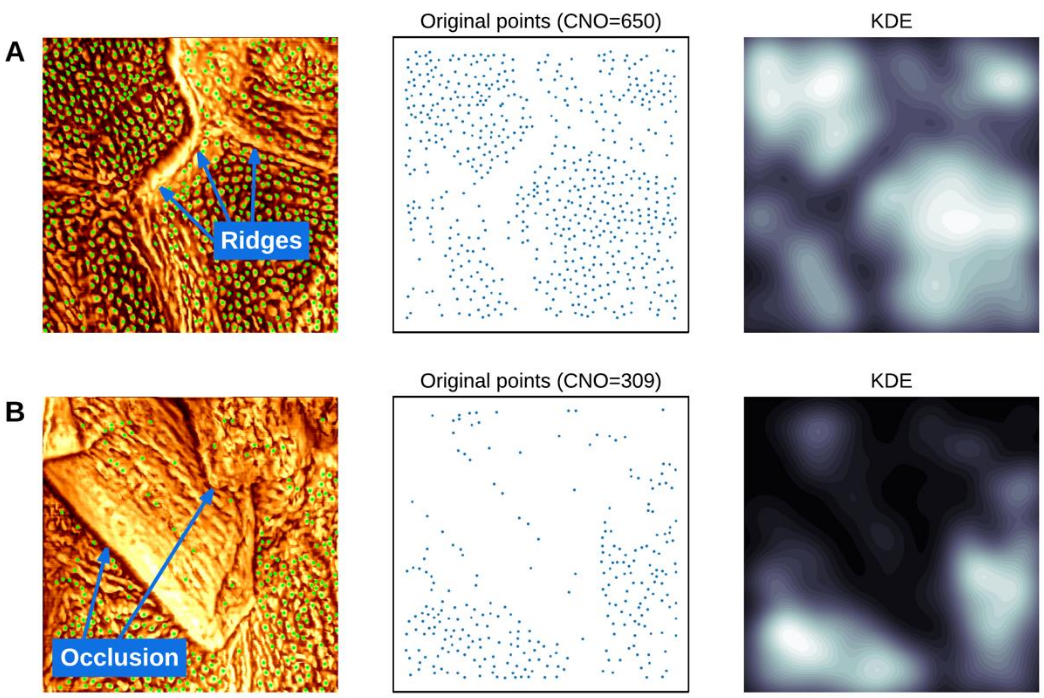

Figure 4. Spatial analysis of CNO distribution using KDE. (A) Corneocyte nanotexture image visualizing the presence of prominent ridges. (B) Corneocyte nanotexture image visualizing an area affected by occlusion. The KDE maps illustrate varying CNO densities, with brighter regions indicating higher densities and darker regions representing lower densities.

Ablation study on KDE

To evaluate the impact of KDE on the variability of CNO density calculations, we conducted an ablation study, comparing results with and without KDE across different AD severity groups. The coefficient of variation (CV) [77] was used as a measure of variability, with lower CV values indicating more stable and consistent density estimates. For each SC sample, the CV was calculated from 10 density estimates derived from its corneocyte nanotexture images. The mean CV for each AD severity group was then determined by averaging the CVs of all samples within the group.

As shown in Figure 5, the application of KDE led to a notable reduction in the CV across most AD groups (G1 to G3), while G4 remained nearly unchanged. Without KDE, the CV values were consistently higher, indicating higher variability in the raw CNO density calculations. Specifically, applying KDE resulted in a reduction of 7.95% in G1 (from 0.440 to 0.405), 18.5% in G2 (from 0.432 to 0.352), 13.0% in G3 (from 0.399 to 0.347), and a slight increase of 1.1% in G4 (from 0.375 to 0.379).

The ablation study demonstrates the effectiveness of KDE in generating more robust density estimates by reducing variability, particularly in AD groups (G1 to G3) with higher CNO presence.

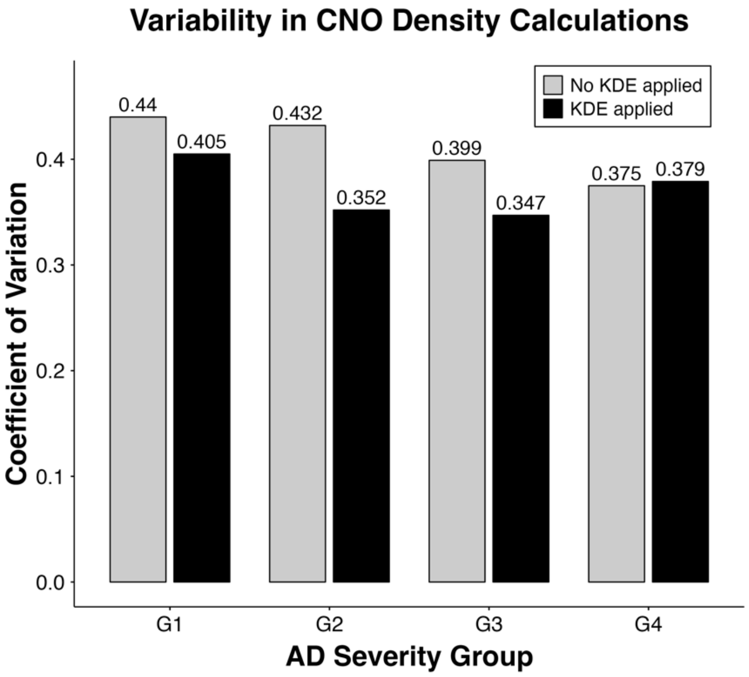

Figure 5. Effect of KDE on the variability in CNO density calculations across AD severity groups (G1, G2, G3, G4). The gray bars indicate CV values without KDE; the black bars show CV values with KDE applied.

Statistical analysis

The mean ECTI scores, derived from the KDE analyses of 10 corneocyte nanotexture images per SC tape, were used for statistical analyses. Each AD group (G1, G2, G3) contributed a total of 30 data points, comprising 15 from lesional and 15 from non-lesional SC samples. In contrast, the healthy control group (G4) contributed 15 data points exclusively from non-lesional SC samples. All images were preprocessed and CNOs were identified using the fine-tuned YOLOv10-L models.

Initially, samples from each AD severity group (G1, G2, G3, G4) underwent the Shapiro-Wilk normality test [78] to assess their data distribution. Given the non-normal distribution observed in most data groups, the Wilcoxon signed-rank test [79] was adopted to determine statistically significant differences between paired samples, focusing on the comparison of lesional and non-lesional SC samples from the same AD patient. In addition, the Wilcoxon rank-sum test [80] was applied to identify significant differences between independent sample groups, specifically among the AD severity groups G1, G2, G3, and G4. Samples with missing data or those that could not be paired for comparison were excluded from the analysis.

Figure 6(A) presents the statistical results using box plots, further subdividing each AD severity group into lesional and non-lesional sampled areas. Overall, the plot reveals a clear trend of increasing ECTI

scores corresponding to the AD severity. Most AD severity groups exhibit significant differences between lesional and non-lesional SC samples, indicating a higher occurrence of CNOs in the lesional skin areas. Additionally, the healthy controls (G4) consistently demonstrate the lowest ECTI scores compared to other AD severity groups. Figure 6(B) presents the statistical analysis of non-lesional SC samples across AD severity groups (G1, G2, G3) compared to the healthy control group (G4), demonstrating significant differences between the AD groups and the healthy controls.

Figure 6(C) provides a comparative analysis of CNO density in non-lesional SC samples calculated over the entire imaging area (20x20 μm2) without using KDE to exclude ineffective regions. The results demonstrate less significant differences between AD severity groups and the healthy control group, particularly being unable to differentiate between mild AD (G1) and healthy controls.

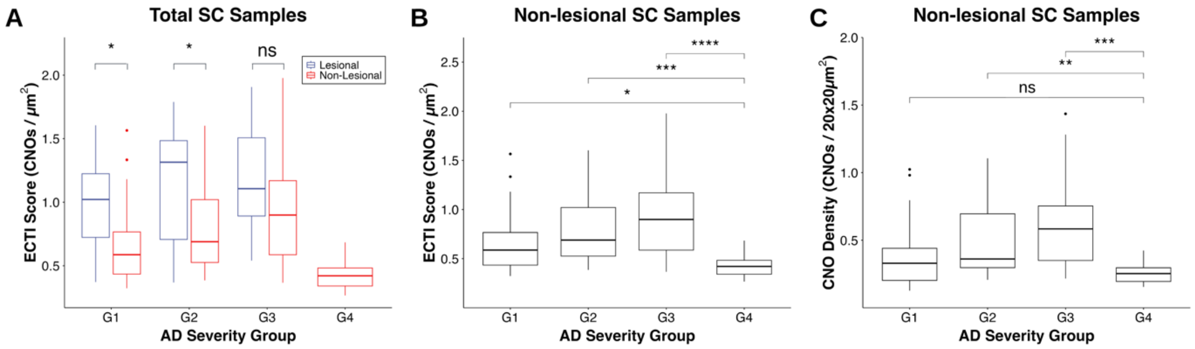

Figure 6. Statistical results of ECTI scores in SC samples from AD patients (n = 15 for both lesional and non-lesional skin areas in each group) and healthy controls (n = 15). (A) Comparison of ECTI scores between lesional and non-lesional SC samples across AD severity groups. (B) ECTI scores for non-lesional SC samples. (C) CNO density in non-lesional SC samples calculated over the entire imaging area (20x20 μm^2) without KDE. Box plot notations: ns → not significant, *p ≤ 0.05, **p ≤ 0.01; AD severity groups according to EASI score: G1 → mild AD, G2 → moderate AD, G3 → severe AD, G4 → healthy controls.

Discussion

The findings of this study demonstrated the potential of corneocyte nanotexture as a reliable biomarker for assessing AD severity, particularly through CNO density calculation. By integrating state-of-the-art deep learning object detectors with spatial analysis algorithms, we proposed the ECTI, an accurate and quantifiable measure for evaluating skin barrier impairment [14]. The ECTI exhibited remarkable robustness in overcoming the inherent challenges of nano-imaging, such as environmental noise and structural occlusions on the corneocyte surface, further enhancing its applicability in clinical settings.

Previous studies revealed significant differences in corneocyte nanotexture between healthy and AD skin samples without specifying the clinical scoring of AD severity, resulting in a lack of in-depth analysis for AD severity assessment [12]. In our study, we conducted statistical analyses of ECTI scores across different AD severity groups (G1, G2, G3, G4), categorized by their EASI scores. The results revealed a clear trend of increasing ECTI scores with higher AD severity and demonstrated significant differences between AD skin samples of varying severity and healthy controls, in both lesional and non-lesional skin areas. This finding aligns with clinical observations of AD severity, offering clinicians a more objective tool for assessing the skin disease.

By leveraging deep learning object detectors, we addressed the limitations of the existing DTI method, which is prone to inaccuracies due to its dependence on fixed geometric criteria for CNO identification. To determine the optimal model architecture for CNO detection, we evaluated the performance of two state-of-the-art object detectors with various scales: YOLOv10-{N, S, M, B, L, X} and RT-DETRv2-{S, M, L, X}. Both models demonstrated robust performance in CNO detection, with the YOLOv10-L model achieving the highest overall accuracy (AP50) of 91.4%. Although RT-DETRv2 exhibited enhanced computational efficiency at comparable model complexities, the YOLOv10 models were more suitable for this study due to their higher detection accuracy.

Furthermore, we applied KDE to perform spatial analysis of CNO distribution. Unlike the DTI, which calculates CNO density across the entire corneocyte nanotexture image (20x20 μm2) without excluding ineffective regions such as ridges and occlusions, our approach selectively excluded these areas to minimize variance in CNO density calculations. This refinement provided a more precise representation of CNO density, enabling us to effectively distinguish between mild AD (G1) and healthy controls (G4) in non-lesional SC samples.

Future work could involve expanding the corneocyte nanotexture database to include a wider range of skin diseases and conditions, providing a more comprehensive and interpretable framework for evaluating skin health through corneocyte nanotexture analysis. Additionally, integrating our findings into clinical practice could substantially improve AD severity assessment by offering an objective and quantifiable evaluation method. Clinicians could utilize corneocyte nanotexture analysis as an accessible and effective tool to monitor disease progression, assess treatment efficacy, and personalize therapeutic interventions for routine clinical use.

This study also acknowledges certain limitations. First, while the sample size in this study is adequate for preliminary analysis, it may not fully capture the variability within the broader population, particularly across diverse ethnic groups and age ranges. Second, the variability in sample collection could lead to inconsistencies. Although a standardized tape-stripping procedure was employed, variations in local eczema severity, exact sampling locations, and individual skin conditions could contribute to discrepancies in the collected SC samples. Moreover, the lack of data on factors such as emollient use, sun exposure, or bathing habits prior to sampling may further affect the results. Finally, as this study focused on AD, the applicability of our approach to other dermatological conditions remains to be validated, necessitating further research to generalize these findings to a wider range of skin diseases.

Conclusion

This study presents a novel methodology that integrates deep learning object detection with spatial analysis to enable robust and accurate CNO density calculation within corneocyte surface topography. The ECTI was introduced as a quantifiable measure for assessing AD severity. Our results revealed significant differences in ECTI scores between SC samples of varying AD severity and healthy controls, in both lesional and non-lesional skin areas, demonstrating its potential as a reliable biomarker for AD assessment. Future work will focus on expanding the corneocyte nanotexture database and exploring the potential of ECTI in broader dermatological applications.

Additional Files

Supplementary Table T1. Hyperparameter settings of YOLOv10.
Supplementary Table T2. Hyperparameter settings of RT-DETRv2.
Supplementary Fig. S1. Training results of YOLOv10-L on the corneocyte nanotexture dataset. The box loss (box) measures the error in predicted bounding box coordinates, the classification loss (cls) quantifies the error in class predictions, and distribution focal loss (dfl) adjusts the bounding box regression by focusing on more challenging examples to improve precision. 'om' denotes evaluation on the training set, and 'oo' indicates evaluation on the validation set.
Supplementary Fig. S2. F1-confidence curve of YOLOv10-L on the corneocyte nanotexture test set. This curve illustrates the relationship between confidence threshold and the F1 score, with the highest F1 score of 0.85 achieved at a confidence threshold of 0.141. This point indicates the optimal balance between precision and recall for the model.

Availability of Source Code and Requirements

• Project name: ECTI Atopic Dermatitis
• Project home page: https://github.com/JenHungWang/ECTI_ Atopic_Dermatitis
• Operating system(s): Platform independent
• Programming language: Python 3.11.4
• Other requirements: Python 3.10+, matplotlib 3.7.2, numpy 1.25.1, opencv-python 4.8.0.74, scipy 1.11.1, scikit-image 0.21.0, scikit-learn 1.3.1, ultralytics 8.2.95, customtkinter 5.2.1
• License: PSF, BSD, Apache, AGPL-3.0
• Workflowhub: https://doi.org/10.48546/workflowhub. workflow.1161.1
• ECTI is registered as a software application on SciCrunch (RRID: SCR_025706) and biotools (biotools:ecti_atopic_dermatitis)

Data Availability

The corneocyte nanotexture dataset, along with the annotations used to train YOLOv10 and RT-DETRv2 object detection models, is available in the GitHub repository [81]. Fine-tuned models and source code can also be downloaded from the same repository. All supporting data and materials are accessible via the *GigaScience* database, GigaDB [82].

Abbreviations

AD: atopic dermatitis; EASI: Eczema Area and Severity Index; SCORAD: SCORing AD; CNO: circular nano-size object; SC: stratum corneum; DTI: Dermal Texture Index; HS-DAFM: high-speed dermal atomic force microscope; NMF: natural moisturizing factor; CNN: convolutional neural network; YOLO: You Only Look Once; NMS: non-maximum suppression; RT-DETR: real-time detection transformer; KDE: kernel density estimator; BW: bandwidth; ECTI: Effective Corneocyte Topographical Index; AP: average precision; IoU: intersection over union; FLOPS: floating-point operations per second; CV: coefficient of variation.

Competing Interests

The authors declare that they have no competing interests.

Funding

This project has received funding from the LEO Foundation under the open competition grant agreement No. LF-OC-20-000370; the Novo Nordisk Foundation under the Pioneer Innovator grant agreement No. NNF22OC0076607; the National Science and Technology Council of Taiwan (NSTC 112-2314-B-002-074-MY3); and the Intelligent Drug Delivery and Sensing using Microcontainers and Nanomechanics (IDUN).

Authors' Contributions

J.-H.W. developed the source code, annotated the dataset, and fine-tuned the deep learning models with support from J.P.; C.-W.D. designed the sample collection protocols and compiled the dataset; I.J., M.O.C., and S.K. provided technical feedback and interpretation of results throughout the development phase; S.S. provided feedback on the clinical applications of the tool; C.-Y.C. and E.E.-T.H. contributed to the critical revision of the manuscript; C.-Y.C., J.P.T., and E.E.-T.H. conceptualized and designed the project. All authors contributed to and approved the final manuscript.

References

1. Langan SM, Irvine AD, Weidinger S. Atopic dermatitis. The Lancet 2020;396(10247):345–360. https://doi.org/10.1016/ S0140-6736(20)31286-1.

2. Barbarot S, Auziere S, Gadkari A, Girolomoni G, Puig L, Simpson EL, et al. Epidemiology of atopic dermatitis in adults: Results from an international survey. Allergy 2018;73:1284–1293. https://doi.org/10.1111/all.13401.

3. Drucker AM, Wang AR, Li WQ, Sevetson E, Block JK, Qureshi AA. The Burden of Atopic Dermatitis: Summary of a Report for the National Eczema Association. Journal of Investigative Dermatology 2017;137(1):26–30. https://doi.org/10.1016/j. jid.2016.07.012.

4. Hanifin JM, Thurston M, Omoto M, Cherill R, Tofte SJ, Graeber M, et al. The eczema area and severity index (EASI): assessment of reliability in atopic dermatitis. Experimental Dermatology 2001;10:11–18. https://doi.org/10.1034/j.1600-0625.2001.100102.x.

5. Kunz B, Oranje AP, Labrèze L, Stalder JF, Ring J, Taïeb A. Clinical Validation and Guidelines for the SCORAD Index: Consensus Report of the European Task Force on Atopic Dermatitis. Dermatology 1997;195:10–19. https://doi.org/10.1159/000245677.

6. Zhao CY, Tran AQT, Lazo-Dizon JP, Kim J, Daniel BS, Venugopal SS, et al. A pilot comparison study of four clinician-rated atopic dermatitis severity scales. British Journal of Dermatology 2015;173:488–497. https://doi.org/10.1111/bjd.13846.

7. Schmitt J, Langan S, Deckert S, Svensson A, von Kobyletzki L, Thomas K, et al. Assessment of clinical signs of atopic dermatitis: A systematic review and recommendation. Journal of Allergy and Clinical Immunology 2013;132:1337–1347. https://doi.org/10.1016/j.jaci.2013.07.008.

8. Thomas KS. EASI does it: a comparison of four eczema severity scales. British Journal of Dermatology 2015;173:316–317. https://doi.org/10.1111/bjd.13967.

9. Hanifin JM, Baghoomian W, Grinich E, Leshem YA, Jacobson M, Simpson EL. The Eczema Area and Severity Index — A Practical Guide. Dermatitis 2022;33:187–192. https://doi.org/10.1097%2FDER.0000000000000895.

10. Riethmuller C, McAleer MA, Koppes SA, Abdayem R, Franz J, Haftek M, et al. Filaggrin breakdown products determine corneocyte conformation in patients with atopic dermatitis. Journal of Allergy and Clinical Immunology 2015;136:1573–1580.e2. https://doi.org/10.1016%2Fj.jaci.2015.04.042.

11. Franz J, Beutel M, Gevers K, Kramer A, Thyssen JP, Kezic S, et al. Nanoscale alterations of corneocytes indicate skin disease. Skin Research and Technology 2016;22:174–180. https://doi.org/10.1111/srt.12247.

12. Engebretsen KA, Bandier J, Kezic S, Riethmüller C, Heegaard NHH, Carlsen BC, et al. Concentration of filaggrin monomers, its metabolites and corneocyte surface texture in individuals with a history of atopic dermatitis and controls. Journal of the European Academy of Dermatology and Venereology 2018;32:796–804. https://doi.org/10.1111/jdv.14801.

13. Riethmüller C. Assessing the skin barrier via corneocyte morphometry. Experimental Dermatology 2018;27:923–930. https://doi.org/10.1111/exd.13741.

14. de Boer FL, van der Molen HF, Kezic S. Epidermal biomarkers of the skin barrier in atopic and contact dermatitis. Contact Dermatitis 2023;89:221–229. https://doi.org/10.1111/cod.14391.

15. Lademann J, Jacobi U, Surber C, Weigmann HJ, Fluhr JW. The tape stripping procedure – evaluation of some critical parameters. European Journal of Pharmaceutics and Biopharmaceutics 2009;72:317–323. https://doi.org/10.1016/j.ejpb.2008.08.008.

16. Liao HS, Akhtar I, Werner C, Slipets R, Pereda J, Wang JH, et al. Open-source controller for low-cost and high-speed atomic force microscopy imaging of skin corneocyte nanotextures. HardwareX 2022;12:e00341. https://doi.org/10.1016/j.ohx.2022.e00341.

17. Chan TC, Wu NL,Wong LS, Cho YT, Yang CY, Yu Y, et al. Taiwanese Dermatological Association consensus for the management of atopic dermatitis: A 2020 update. Journal of the Formosan Medical Association 2021;120(1):429–442. http://dx.doi.org/10.1016/j.jfma.2020.06.008.

18. Hajian-Tilaki K. Sample size estimation in diagnostic test studies of biomedical informatics. Journal of Biomedical Informatics 2014;48:193–204. http://dx.doi.org/10.1016/j.jbi.2014.02.013.

19. Heinisch O. Cochran,W. G.: Sampling Techniques, 2. Aufl. John Wiley and Sons, New York, London 1963. Preis s. Biometrische Zeitschrift 1965;7(3):203–203. http://dx.doi.org/10.1002/bimj.19650070312.

20. Dapic I, Jakasa I, Yau NLH, Kezic S, Kammeyer A. Evaluation of an HPLC Method for the Determination of Natural Moisturizing Factors in the Human Stratum Corneum. Analytical Letters 2013;46:2133–2144. https://doi.org/10.1080/00032719.2013.789881.

21. Inoue T, Kuwano T, Uehara Y, Yano M, Oya N, Takada N, et al. Non-invasive human skin transcriptome analysis using mRNA in skin surface lipids. Communications Biology 2022;5:215. https://doi.org/10.1111/jdv.18173.

22. Kezic S, Kammeyer A, Calkoen F, Fluhr JW, Bos JD. Natural moisturizing factor components in the stratum corneum as biomarkers of filaggrin genotype: evaluation of minimally invasive methods. British Journal of Dermatology 2009;161:1098– 1104. https://doi.org/10.1111/j.1365-2133.2009.09342.x.

23. Hwu EET, Boisen A. Hacking CD/DVD/Blu-ray for Biosensing. ACS Sensors 2018;3(7):1222–1232. http://dx.doi.org/10. 1021/acssensors.8b00340.

24. Kienberger F, Pastushenko VP, Kada G, Puntheeranurak T, Chtcheglova L, Riethmueller C, et al. Improving the contrast of topographical AFM images by a simple averaging filter. Ultramicroscopy 2006;106:822–828. https://doi.org/10.1016/j.ultramic.2005.11.013.

25. Kimori Y. Mathematical morphology-based approach to the enhancement of morphological features in medical images. Journal of Clinical Bioinformatics 2011;1:33. https://doi.org/10.1186/2043-9113-1-33.

26. Gedraite ES, Hadad M. Investigation on the effect of a Gaussian Blur in image filtering and segmentation. In: Proceedings ELMAR-2011; 2011. p. 393–396. https://ieeexplore.ieee.org/document/6044249.

27. Eaton P, West P. Atomic Force Microscopy. Oxford University Press; 2010. http://dx.doi.org/10.1093/acprof:oso/ 9780199570454.001.0001.

28. Kubo S, Umeda K, Kodera N, Takada S. Removing the parachuting artifact using two-way scanning data in high-speed atomic force microscopy. Biophysics and Physicobiology 2023;20(1). http://dx.doi.org/10.2142/biophysico.bppb-v20.0006.

29. Toet A. Adaptive multi-scale contrast enhancement through non-linear pyramid recombination. Pattern Recognition Letters 1990;11(11):735–742. http://dx.doi.org/10.1016/ 0167-8655(90)90092-G.

30. Haralick RM, Sternberg SR, Zhuang X. Image Analysis Using Mathematical Morphology. IEEE Transactions on Pattern Analysis and Machine Intelligence 1987;PAMI-9:532–550. https://doi.org/10.1109/TPAMI.1987.4767941.

31. Oh J, Hwang H. Feature enhancement of medical images using morphology-based homomorphic filter and differential evolution algorithm. International Journal of Control, Automation and Systems 2010;8:857–861. https://doi.org/10.1007/s12555-010-0418-y.

32. Jia X, Tong Y, Qiao H, Li M, Tong J, Liang B. Fast and accurate object detector for autonomous driving based on improved YOLOv5. Scientific Reports 2023;13(1). http://dx.doi.org/10.1038/s41598-023-36868-w.

33. Bogdoll D, Nitsche M, Zollner JM. Anomaly Detection in Autonomous Driving: A Survey. In: 2022 IEEE/CVF Conference on Computer Vision and Pattern Recognition Workshops (CVPRW) IEEE; 2022. http://dx.doi.org/10.1109/CVPRW56347.2022.00495.

34. Sobek J, Medina Inojosa JR, Medina Inojosa BJ, Rassoulinejad-Mousavi SM, Conte GM, Lopez-Jimenez F, et al. MedYOLO: A Medical Image Object Detection Framework. Journal of Imaging Informatics in Medicine 2024;http://dx.doi.org/10.1007/s10278-024-01138-2.

35. Shou Y, Meng T, Ai W, Xie C, Liu H, Wang Y. Object Detection in Medical Images Based on Hierarchical Transformer and Mask Mechanism. Computational Intelligence and Neuroscience 2022;2022:1–12. http://dx.doi.org/10.1155/2022/5863782.

36. Girshick R. Fast R-CNN. In: 2015 IEEE International Conference on Computer Vision (ICCV) IEEE; 2015. http://dx.doi.org/10. 1109/ICCV.2015.169.

37. He K, Gkioxari G, Dollar P, Girshick R. Mask R-CNN. IEEE Transactions on Pattern Analysis and Machine Intelligence 2020;42(2):386–397. http://dx.doi.org/10.1109/TPAMI.2018.2844175.

38. Redmon J, Divvala S, Girshick R, Farhadi A. You Only Look Once: Unified, Real-Time Object Detection. In: 2016 IEEE Conference on Computer Vision and Pattern Recognition (CVPR) IEEE; 2016. http://dx.doi.org/10.1109/CVPR.2016.91.

39. Redmon J, Farhadi A. YOLO9000: Better, Faster, Stronger. In: 2017 IEEE Conference on Computer Vision and Pattern Recognition (CVPR) IEEE; 2017. http://dx.doi.org/10.1109/ CVPR.2017.690.

40. Redmon J, Farhadi A, YOLOv3: An Incremental Improvement; Computer vision and pattern recognition 2018. 1804:1-6. https://doi.org/10.48550/arXiv.1804.02767

41. Carion N, Massa F, Synnaeve G, Usunier N, Kirillov A, Zagoruyko S. In: End-to-End Object Detection with Transformers Springer International Publishing; 2020. p. 213–229. http://dx.doi.org/10.1007/978-3-030-58452-8_13.

42. Zhu X, Su W, Lu L, Li B, Wang X, Dai J, Deformable DETR: Deformable Transformers for End-to-End Object Detection; arXiv 2021. https://doi.org/10.48550/arXiv.2010.04159.

43. Zhang H, Li F, Liu S, Zhang L, Su H, Zhu J, et al., DINO: DETR with Improved DeNoising Anchor Boxes for End-to-End Object Detection; arXiv 2022. https://doi.org/10.48550/arXiv.2203.03605.

44. Li F, Zhang H, Liu S, Guo J, Ni LM, Zhang L. DN-DETR: Accelerate DETR Training by Introducing Query DeNoising. In: 2022 IEEE/CVF Conference on Computer Vision and Pattern Recognition (CVPR) IEEE; 2022. http://dx.doi.org/10.1109/ CVPR52688.2022.01325.

45. Liu S, Li F, Zhang H, Yang X, Qi X, Su H, et al., DAB-DETR: Dynamic Anchor Boxes are Better Queries for DETR; arXiv 2022. https://doi.org/10.48550/arXiv.2201.12329.

46. Meng D, Chen X, Fan Z, Zeng G, Li H, Yuan Y, et al. Conditional DETR for Fast Training Convergence. In: 2021 IEEE/CVF International Conference on Computer Vision (ICCV) IEEE; 2021. http://dx.doi.org/10.1109/ICCV48922.2021.00363.

47. Wang Y, Zhang X, Yang T, Sun J. Anchor DETR: Query Design for Transformer-Based Detector. Proceedings of the AAAI Conference on Artificial Intelligence 2022;36(3):2567–2575. http://dx.doi.org/10.1609/aaai.v36i3.20158.

48. Bochkovskiy A, Wang CY, Liao HYM, YOLOv4: Optimal Speed and Accuracy of Object Detection; arXiv 2020. https://doi.org/10.48550/arXiv.2004.10934.

49. Ge Z, Liu S, Wang F, Li Z, Sun J, YOLOX: Exceeding YOLO Series in 2021; arXiv 2021. https://doi.org/10.48550/arXiv.2107.08430.

50. Wang CY, Bochkovskiy A, Liao HYM. Scaled-YOLOv4: Scaling Cross Stage Partial Network. In: 2021 IEEE/CVF Conference on Computer Vision and Pattern Recognition (CVPR) IEEE; 2021. http://dx.doi.org/10.1109/CVPR46437.2021.01283.

51. Chen Y, Yuan X, Wu R, Wang J, Hou Q, Cheng MM, YOLO-MS: Rethinking Multi-Scale Representation Learning for Real-time Object Detection; arXiv 2023. https://doi.org/10.48550/arXiv.2308.05480.

52. Huang L, Li W, Shen L, Fu H, Xiao X, Xiao S, YOLOCS: Object Detection based on Dense Channel Compression for Feature Spatial Solidification; arXiv 2023. https://doi.org/10.48550/arXiv.2305.04170.

53. Li C, Li L, Geng Y, Jiang H, Cheng M, Zhang B, et al., YOLOv6 v3.0: A Full-Scale Reloading; arXiv 2023. https://doi.org/10.48550/arXiv.2301.05586.

54. Wang C, He W, Nie Y, Guo J, Liu C, Han K, et al., Gold-YOLO: Efficient Object Detector via Gather-and-Distribute Mechanism; arXiv 2023. https://doi.org/10.48550/arXiv.2309.11331.

55. Wang CY, Bochkovskiy A, Liao HYM. YOLOv7: Trainable Bag-of-Freebies Sets New State-of-the-Art for Real-Time Object Detectors. In: 2023 IEEE/CVF Conference on Computer Vision and Pattern Recognition (CVPR) IEEE; 2023. http://dx.doi. org/10.1109/CVPR52729.2023.00721.

56. Varghese R, M S. YOLOv8: A Novel Object Detection Algorithm with Enhanced Performance and Robustness. In: 2024 International Conference on Advances in Data Engineering and Intelligent Computing Systems (ADICS) IEEE; 2024. http://dx.doi.org/10.1109/ADICS58448.2024.10533619.

57. Wang CY, Yeh IH, Liao HYM, YOLOv9: Learning What You Want to Learn Using Programmable Gradient Information; arXiv 2024. https://doi.org/10.48550/arXiv.2402.13616.

58. Wang A, Chen H, Liu L, Chen K, Lin Z, Han J, et al., YOLOv10: Real-Time End-to-End Object Detection; arXiv 2024. https://doi.org/10.48550/arXiv.2405.14458.

59. Li C, Li L, Jiang H, Weng K, Geng Y, Li L, et al., YOLOv6: A Single-Stage Object Detection Framework for Industrial Applications; arXiv 2022. https://doi.org/10.48550/arXiv.2209.02976.

60. Rahman S, Rony JH, Uddin J, Samad MA. Real-Time Obstacle Detection with YOLOv8 in a WSN Using UAV Aerial Photography. Journal of Imaging 2023;9(10):216. http://dx.doi.org/ 10.3390/jimaging9100216.

61. Khare O, Gandhi S, Rahalkar A, Mane S. YOLOv8-Based Visual Detection of Road Hazards: Potholes, Sewer Covers, and Manholes. In: 2023 IEEE Pune Section International Conference (PuneCon) IEEE; 2023. http://dx.doi.org/10.1109/PuneCon58714.2023.10449999.

62. Wang CY, Liao HYM, YOLOv1 to YOLOv10: The fastest and most accurate real-time object detection systems; arXiv 2024. https://doi.org/10.48550/arXiv.2408.09332.

63. Hussain M, YOLOv5, YOLOv8 and YOLOv10: The Go-To Detectors for Real-time Vision; arXiv 2024. https://doi.org/10.48550/arXiv.2407.02988.

64. Sun P, Jiang Y, Xie E, Shao W, Yuan Z, Wang C, et al. What Makes for End-to-End Object Detection? In: Proceedings of the 38th International Conference on Machine Learning, vol. 139 of Proceedings of Machine Learning Research PMLR; 2021. p. 9934–9944. https://proceedings.mlr.press/v139/sun21b.html.

65. Zhao Y, Lv W, Xu S, Wei J, Wang G, Dang Q, et al., DETRs Beat YOLOs on Real-time Object Detection; arXiv 2024. https://doi.org/10.48550/arXiv.2304.08069.

66. Lv W, Zhao Y, Chang Q, Huang K, Wang G, Liu Y, RT-DETRv2: Improved Baseline with Bag-of-Freebies for Real-Time Detection Transformer; arXiv 2024. https://doi.org/10.48550/arXiv.2407.17140.

67. Xu M, Yoon S, Fuentes A, Park DS. A Comprehensive Survey of Image Augmentation Techniques for Deep Learning. Pattern Recognition 2023;137:109347. http://dx.doi.org/10.1016/j.patcog.2023.109347.

68. Shorten C, Khoshgoftaar TM. A survey on Image Data Augmentation for Deep Learning. Journal of Big Data 2019;6(1). http://dx.doi.org/10.1186/s40537-019-0197-0.

69. Chen YC. A tutorial on kernel density estimation and recent advances. Biostatistics & Epidemiology 2017;1(1):161–187. http://dx.doi.org/10.1080/24709360.2017.1396742.

70. Węglarczyk S. Kernel density estimation and its application. ITM Web of Conferences 2018;23:00037. http://dx.doi.org/ 10.1051/itmconf/20182300037.

71. Heidenreich NB, Schindler A, Sperlich S. Bandwidth selection for kernel density estimation: a review of fully automatic selectors. AStA Advances in Statistical Analysis 2013;97(4):403–433. http://dx.doi.org/10.1007/s10182-013-0216-y.

72. Everingham M, Eslami SMA, Van Gool L, Williams CKI, Winn J, Zisserman A. The Pascal Visual Object Classes Challenge: A Retrospective. International Journal of Computer Vision 2014;111(1):98–136. http://dx.doi.org/10.1007/s11263-014-0733-5.

73. Russakovsky O, Deng J, Su H, Krause J, Satheesh S, Ma S, et al. ImageNet Large Scale Visual Recognition Challenge. International Journal of Computer Vision 2015;115(3):211–252. http://dx.doi.org/10.1007/s11263-015-0816-y.

74. Everingham M, Van Gool L, Williams CKI, Winn J, Zisserman A. The Pascal Visual Object Classes (VOC) Challenge. International Journal of Computer Vision 2009;88(2):303–338. http://dx.doi.org/10.1007/s11263-009-0275-4.

75. Zhou Y, Yang K. Exploring TensorRT to Improve Real-Time Inference for Deep Learning. In: 2022 IEEE 24th Int Conf on High Performance Computing & Communications; 8th Int Conf on Data Science & Systems; 20th Int Conf on Smart City; 8th Int Conf on Dependability in Sensor, Cloud & Big Data Systems & Application (HPCC/DSS/SmartCity/DependSys) IEEE; 2022. http://dx.doi.org/10.1109/HPCC-DSS-SmartCity-DependSys57074.2022.00299.

76. Ganguly P, Methani NS, Khapra MM, Kumar P. A Systematic Evaluation of Object Detection Networks for Scientific Plots. Proceedings of the AAAI Conference on Artificial Intelligence 2021;35(2):1379–1387. http://dx.doi.org/10.1609/aaai.v35i2.16227.

77. Reed GF, Lynn F, Meade BD. Use of Coefficient of Variation in Assessing Variability of Quantitative Assays. Clinical and Vaccine Immunology 2003;10(6):1162–1162. http://dx.doi.org/10.1128/CDLI.10.6.1162.2003.

78. SHAPIRO SS, WILK MB. An analysis of variance test for normality (complete samples). Biometrika 1965;52(3–4):591–611. http://dx.doi.org/10.1093/biomet/52.3-4.591.

79. Rey D, Neuhäuser M. In: Wilcoxon-Signed-Rank Test Springer Berlin Heidelberg; 2011. p. 1658–1659. http://dx.doi.org/10. 1007/978-3-642-04898-2_616.

80. Fay MP, Proschan MA. Wilcoxon-Mann-Whitney or t-test? On assumptions for hypothesis tests and multiple interpretations of decision rules. Statistics Surveys 2010;4(none). http://dx. doi.org/10.1214/09-SS051.

81. Wang JH, Pereda J, Hwu EET, Source code: Stratum corneum nanotexture feature detection using deep learning and spatial analysis; 2024. https://github.com/JenHungWang/ECTI_ Atopic_Dermatitis, Last accessed on 2024-09-03.

82. Wang J; Pereda J; Du C; Chu C; Christensen MO; Kezic S; Jakasa I; Thyssen JP; Satheesh S; Hwu EE. Supporting data for "Stratum corneum nanotexture feature detection using deep learning and spatial analysis: a non-invasive tool for skin barrier assessment" GigaScience Database 2024. https://doi.org/10.5524/102604

Click here to access/download;Figure;Paper Figure 1.png

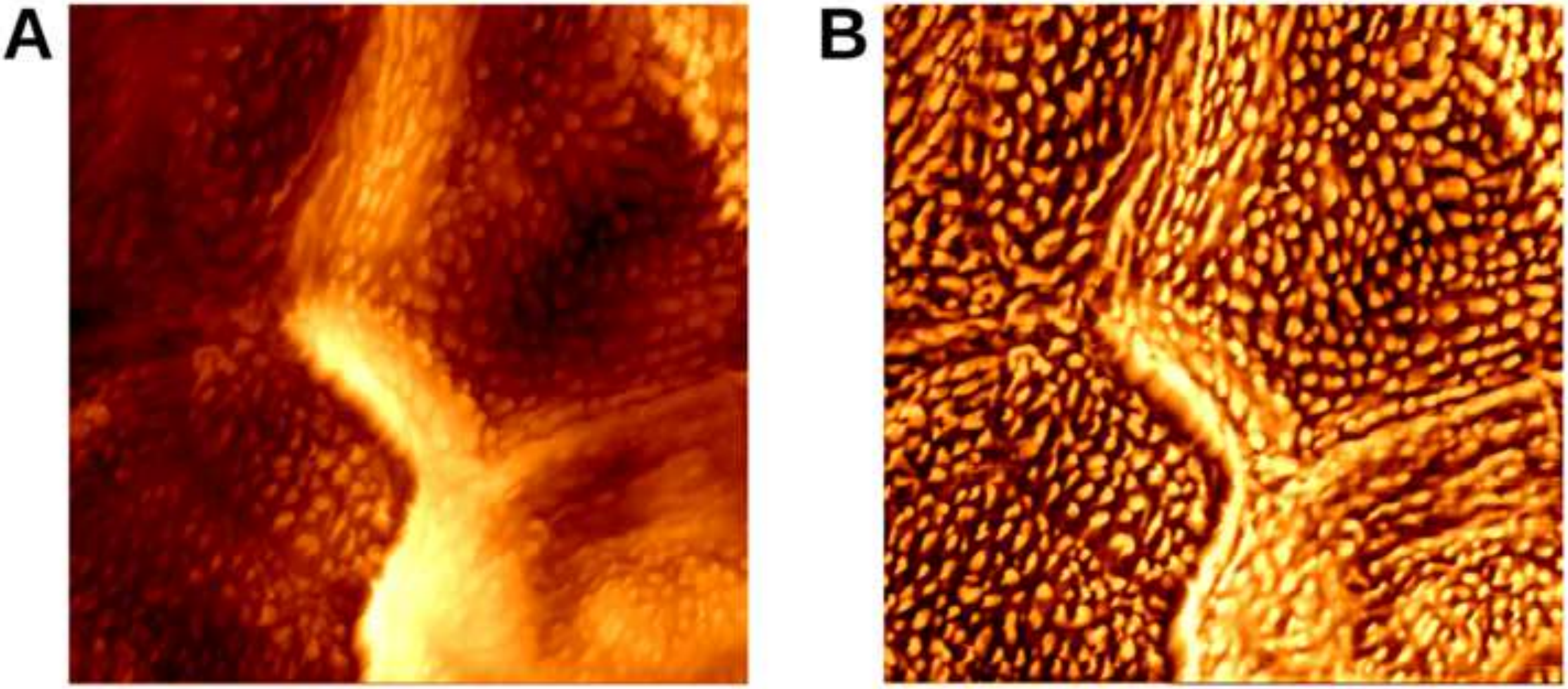

Click here to access/download;Figure;Paper Figure 2.png

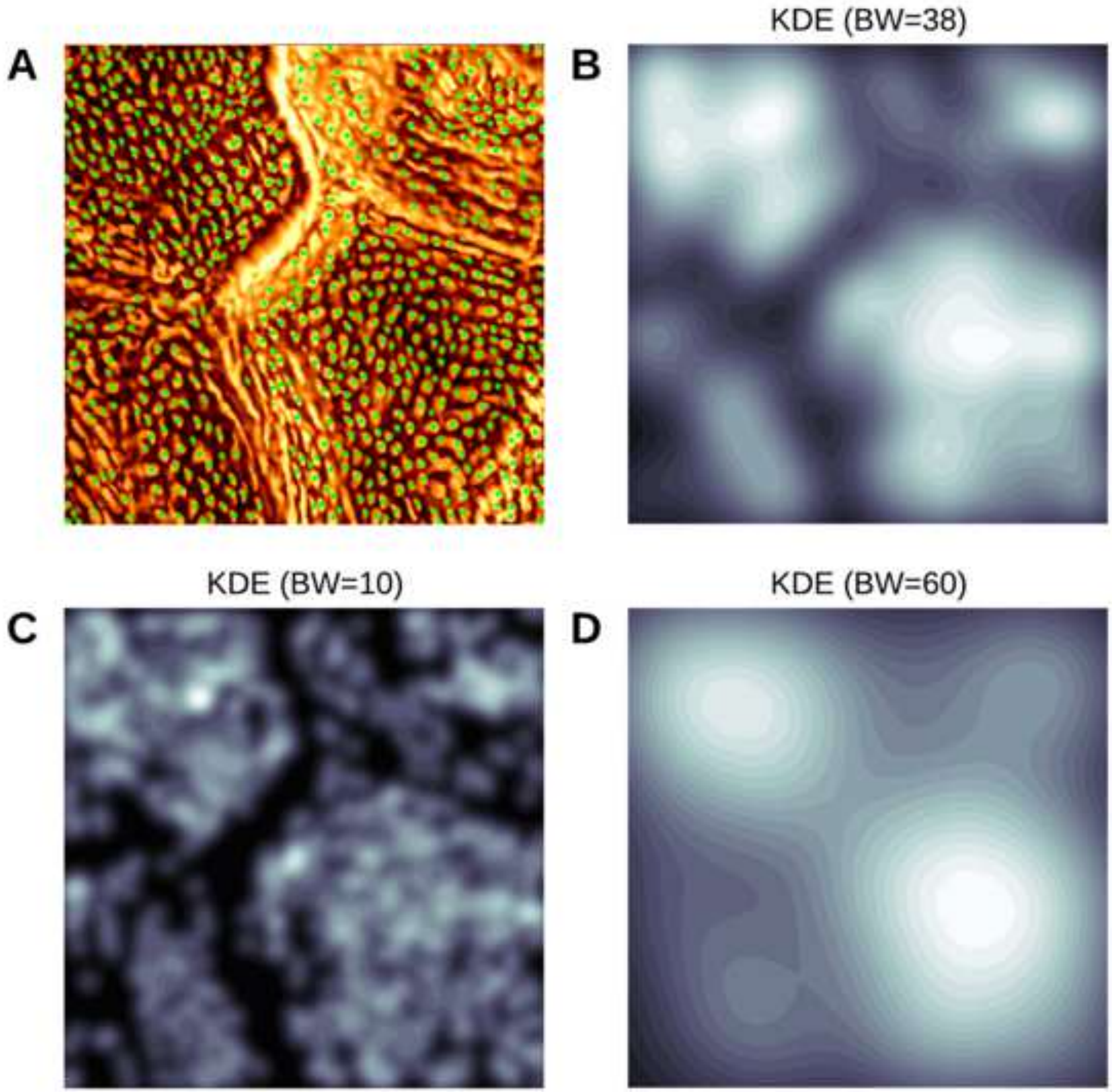

Click here to access/download;Figure;Paper Figure 3.png

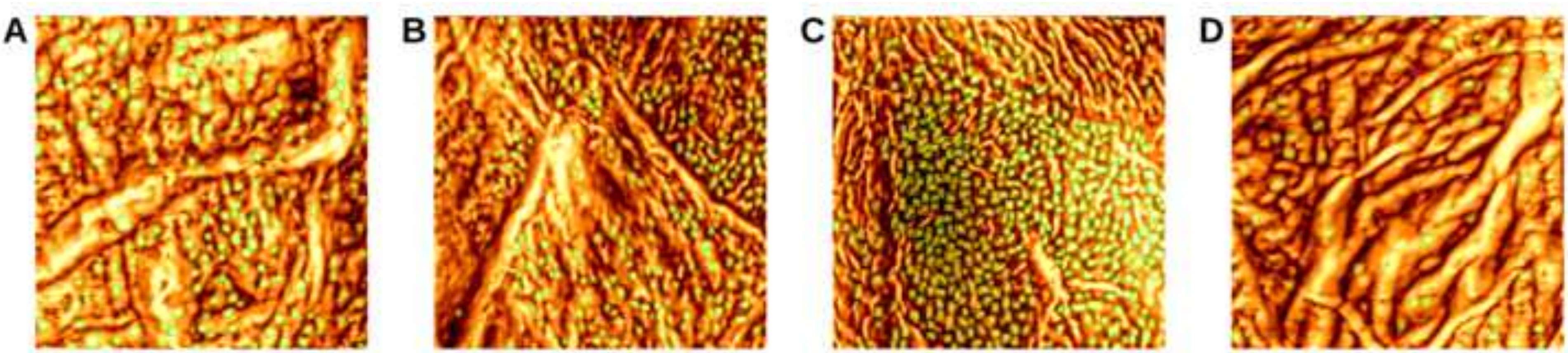

Click here to access/download;Figure;Paper Figure 4.png

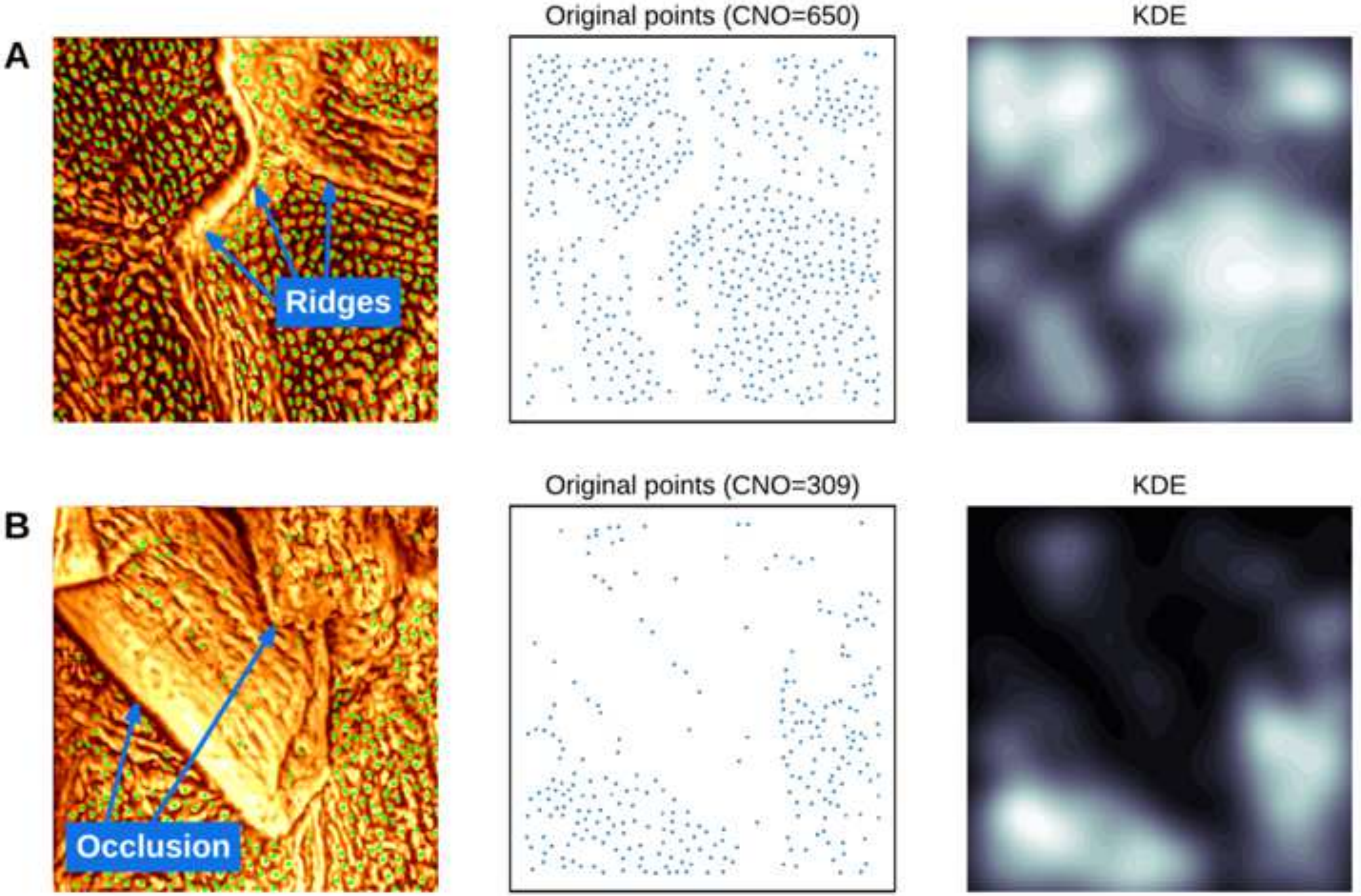

Click here to access/download;Figure;Paper Figure 5.png

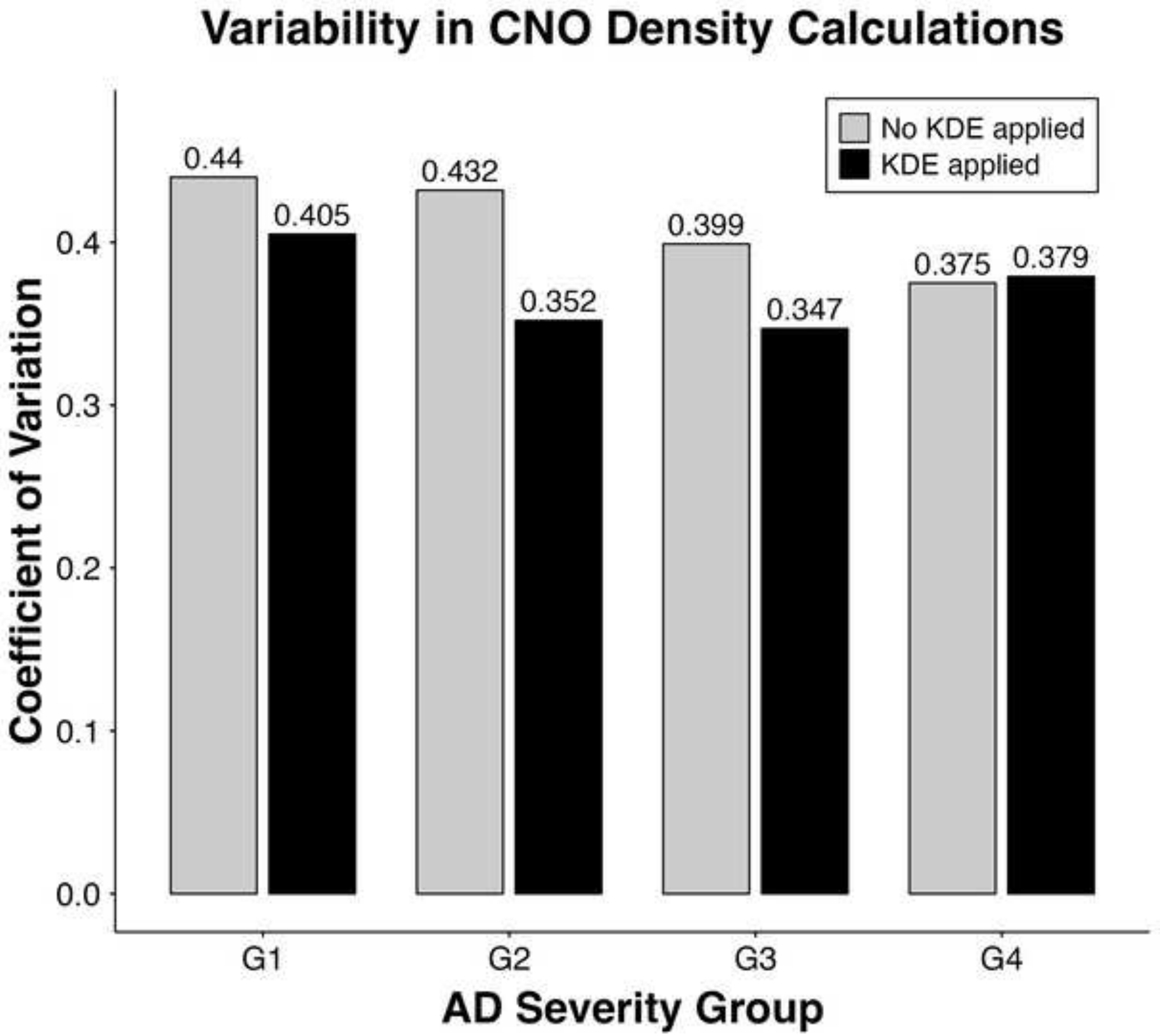

Click here to access/download;Figure;Paper Figure 6.png

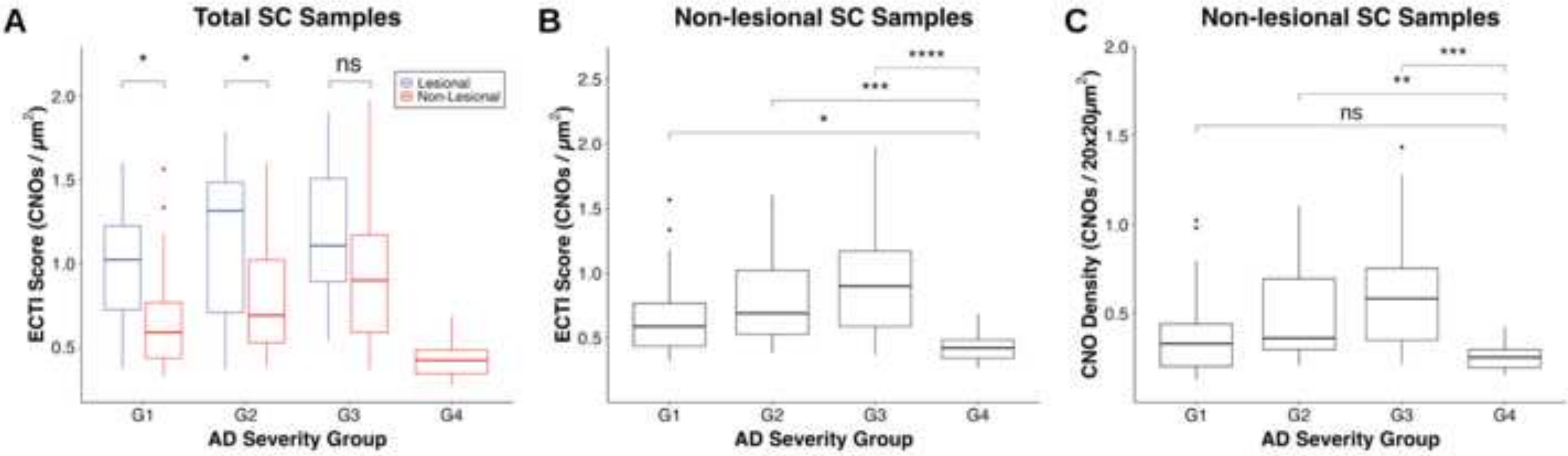

(GIGA)n SCIENCE
Click here

Click here to access/download
Supplementary Material
GIGA-D-24-00100_Supplementary Figures and Tables.pdf

Personal Cover Click here to access/download;Personal Cover;GIGA-D-24-00100_Resubmission Cover Letter.pdf

Dear Editor,

We are pleased to submit the revised version of our manuscript "Stratum corneum nanotexture feature detection using deep learning and spatial analysis: a non-invasive tool for skin barrier assessment" (GIGA-D-24-00100) for consideration by GigaScience. We greatly appreciate the thoughtful feedback provided by you and the reviewers, which has helped us significantly improve our manuscript.

In response to the reviewers' comments, we have made substantial revisions to the manuscript, including:

1. Updating our object detection models to include the latest YOLOv10 and RT-DETRv2, providing a more comprehensive comparison of state-of-the-art deep learning approaches.
2. Adding an ablation study on the kernel density estimator (KDE) to demonstrate the robustness of our approach in calculating CNO density.
3. Revising our method for selecting the confidence threshold based on the F1-confidence curve, ensuring an optimal balance between precision and recall.
4. Providing a more detailed explanation of image preprocessing techniques and hyperparameter selection for the deep learning models.
5. Expanding the discussion on potential limitations and sources of error in the study.
6. Clarifying the theoretical support for our sample size estimation and scanning range selection.

We believe these revisions have substantially strengthened our manuscript and addressed all the concerns raised by the reviewers. The updated manuscript now offers a more comprehensive and rigorous analysis of our proposed Effective Corneocyte Topographical Index (ECTI) for assessing atopic dermatitis severity.

All authors have reviewed and approved this revised version. We would like to add Prof. Chia-Yu Chu as second corresponding author for the dermatology part.

We have also ensured that our software application is registered in the bio.tools and SciCrunch.org databases, receiving RRID (SCR_025706) and biotoolsID (ecti_atopic_dermatitis) identifiers. Additionally, our computational workflow has been registered in workflowhub.eu (https://doi.org/10.48546/workflowhub.workflow.1161.1).

We appreciate your consideration and look forward to your response.

Sincerely,

Edwin En-Te Hwu

On behalf of all co-authors
