# Supplementary material for: Stratum corneum nanotexture feature detection using deep learning and spatial analysis: a noninvasive tool for skin barrier assessment
Source: Gigascience. 2024 Dec 4;13:giae095. doi: 10.1093/gigascience/giae095 (PMC11629979; doi:10.1093/gigascience/giae095)
Supplement: giae095_Response_to_Reviewer_Comments_Original_Submission [file giae095_response_to_reviewer_comments_original_submission.pdf]

Author's Response To Reviewer Comments

Reviewer #1:
1. The paper utilizes YOLO object detection methods, including the recently proposed YOLOv8 and YOLOv9. Table 1 demonstrates that achieving optimal performance across all metrics is not feasible with a singular approach. Could you elaborate on the reasons behind this phenomenon?

Response:
We thank the reviewer's comment. The average precision (AP) score is a widely recognized metric for evaluating object detection models [1,2], as it provides a comprehensive assessment by combining recall, precision, and intersection over union (IoU) into a single measure. However, precision and recall often exhibit an inverse relationship, where improving one typically compromises the other. Consequently, achieving optimal performance across all metrics with a single approach is not feasible due to these inherent trade-offs.
Since precision and recall values are typically omitted in evaluations of object detection models [3,4], we focused on AP50 and AP50-95 scores [5] in the revised manuscript. This decision provides a clearer and more concise representation of our models' performance, enabling readers to more accurately assess the results.
Modified section in the manuscript: Analyses - Comparative analysis of deep learning object detector

2. Have you explored other object detection methods, such as two-stage methods like Mask R-CNN and one-stage methods like SSD, in addition to YOLO?

Response:
We appreciate the reviewer's suggestion. To offer a more comprehensive comparison for selecting object detection models in identifying circular nano-size objects (CNOs), we also trained the recently proposed end-to-end Transformer-based detectors (RT-DETR) [4,6] alongside the latest YOLOv10 models [3]. Following the reviewer's suggestion, we compare the performance of these state-of-the-art object detectors, allowing for a more informed decision in selecting the most effective model for our specific application.
Modified section in the manuscript: Material and Methods - Training deep learning object detectors for CNO detection

3. For experimental analysis, it is better to present quantitative visualization results of comparative methods, which can be shown in a figure.

Response:
We have now included an ablation study on kernel density estimation (KDE). This analysis quantifies the effect of KDE on the variability of CNO density calculations across AD severity groups, demonstrating the robustness of our approach.
Modified section in the manuscript: Analyses - Ablation study on KDE

4. Why did the authors choose a confidence threshold of 0.3? Have they experimented with other threshold values?

Response:
We thank the reviewer's suggestion. In the revised manuscript, we have updated the method for selecting the confidence threshold. We now choose the confidence threshold based on the F1-confidence curve [7] to ensure optimal balance between precision and recall. This approach allows us to determine the threshold that maximizes the F1 score, providing a more reliable criterion for evaluating model performance.
Modified section in the manuscript: Analyses - Qualitative results

5. Recently, YOLOv10 has been proposed as an improvement over YOLOv9 (https://github.com/THU-MIG/yolov10). The authors might consider evaluating detection performance using YOLOv10.

Response:
We followed the reviewer’s comment then trained and evaluated the latest YOLOv10 models for CNO detection, comparing their performance with Transformer-based models (RT-DETRv2) [4,6] in the revised manuscript.
Modified section in the manuscript: Analyses - Comparative analysis of deep learning object detectors

Reviewer #2:
1. What is the theoretical support for the estimation of sample size?

Response:
The sample size was estimated using Cochran’s formula, based on a 6.7% prevalence of AD in the Taiwanese population [8]. The estimation indicated that at least 41 participants would be required to achieve an 80% confidence level, ensuring the true value falls within ±5% of the measured value [9,10]. In our study, we recruited a total of 45 AD patients and 15 healthy controls (≥18 years) in Taiwan, satisfying the calculated requirements.
The selection of the 20x20 μm² scanning range is based on the article "Nanoscale alterations of corneocytes indicate skin disease" (https://doi.org/10.1111/srt.12247), which details the typical dimensions of CNO (273 nm height and 305 nm width). This ensures that the chosen scanning range is appropriate for capturing the relevant nanoscale features.
Additionally, to calibrate the high-speed dermal atomic force microscope (HS-DAFM) scanner, a piece of a data track layer (ca. 1x1 cm²) from a rewritable DVD was used as the calibration sample [11]. The DVD data tracks are characterized by a fixed period of 740 nm and a defined depth of 160 nm, allowing precise scanner calibration by measuring them [12]. This ensured that the corneocyte nanotexture images were captured at a resolution of 512x512 pixels over an imaging area of 20x20 μm², providing consistency in the quality and scale of the measurements.
Modified section in the manuscript: Material and Methods - Stratum corneum sample collection / Corneocyte surface topography dataset

2. Please provide a more detailed introduction to the image preprocessing techniques and the selection of hyperparameters for the deep models. What are the considerations behind each choice? Please also provide the corresponding code for review.

Response:
The revised manuscript includes a more detailed explanation of the image preprocessing techniques, and the corresponding code is available in our GitHub repository under utils/img_preprocessing.py, with detailed comments for clarity. For hyperparameter selection, we followed the train-from-scratch settings outlined in [3,4] for both the YOLOv10 and RT-DETRv2 models, ensuring consistency with established protocols. Detailed hyperparameter settings for each model are provided in the Supplementary Table S1. and S2. for further reference.
Modified section in the manuscript: Material and Methods - Image preprocessing / Training deep learning object detectors for CNO detection

3. Please include additional comparisons with existing methods or biomarkers to better demonstrate the novelty and superiority of the proposed approach.

Response:
We appreciate the reviewer’s suggestion. In the revised manuscript, we have included an ablation study comparing the CNO density calculated using the kernel density estimator (KDE) with the traditional method of calculating density over the entire area, similar to the Dermal Texture Index (DTI) [13]. This analysis quantifies the effect of KDE on the variability of CNO density calculations across AD severity groups, demonstrating the robustness of our approach.
In addition, Figure 6(B) and (C) present statistical analyses of CNO density calculated with and without the application of KDE. Figure 6(B) shows that KDE more effectively distinguishes between mild AD and healthy controls in non-lesional SC samples, whereas the analysis without KDE fails to achieve this distinction, as shown in Figure 6(C).

Modified section in the manuscript: Analyses - Ablation study on KDE

4. The discussion on the potential limitations and sources of error in the study could be more extensive.

Response:
In response to the reviewer's feedback, we have expanded the discussion on the potential limitations and sources of error in the revised manuscript. Key limitations include the variability in sample collection due to differences in local eczema severity, exact sampling locations, and individual skin conditions, which could introduce inconsistencies in the results. We also acknowledge that the generalizability of the Effective Corneocyte Topographical Index (ECTI) to other dermatological conditions beyond AD remains to be validated, necessitating further research.
Modified section in the manuscript: Discussion

References

[1] Everingham M, Eslami SMA, Van Gool L, Williams CKI, Winn J, Zisserman A. The Pascal Visual Object Classes Challenge: A Retrospective. International Journal of Computer Vision 2014;111(1):98–136. http://dx.doi.org/10.1007/s11263-014-0733-5.
[2] Russakovsky O, Deng J, Su H, Krause J, Satheesh S, Ma S, et al. ImageNet Large Scale Visual Recognition Challenge. International Journal of Computer Vision 2015;115(3):211–252. http://dx.doi.org/10.1007/s11263-015-0816-y.
[3] Wang A, Chen H, Liu L, Chen K, Lin Z, Han J, et al., YOLOv10: Real-Time End-to-End Object Detection; 2024. https://arxiv.org/abs/2405.14458.
[4] Zhao Y, LvW, Xu S,Wei J,Wang G, Dang Q, et al., DETRs Beat YOLOs on Real-time Object Detection; 2024. https://arxiv.org/abs/2304.08069.
[5] EveringhamM, Van Gool L,Williams CKI,Winn J, Zisserman A. The Pascal Visual Object Classes (VOC) Challenge. International Journal of Computer Vision 2009;88(2):303–338. http://dx.doi.org/10.1007/s11263-009-0275-4.
[6] LvW, Zhao Y, Chang Q, Huang K,Wang G, Liu Y, RT-DETRv2: Improved Baseline with Bag-of-Freebies for Real-Time Detection Transformer; 2024. https://arxiv.org/abs/2407.17140.
[7] Ganguly P, Methani NS, Khapra MM, Kumar P. A Systematic Evaluation of Object Detection Networks for Scientific Plots. Proceedings of the AAAI Conference on Artificial Intelligence 2021;35(2):1379–1387. http://dx.doi.org/10.1609/aaai.v35i2.16227.
[8] Chan TC, Wu NL,Wong LS, Cho YT, Yang CY, Yu Y, et al. Taiwanese Dermatological Association consensus for the management of atopic dermatitis: A 2020 update. Journal of the Formosan Medical Association 2021;120(1):429–442. http://dx.doi.org/10.1016/j.jfma.2020.06.008.
[9] Hajian-Tilaki K. Sample size estimation in diagnostic test studies of biomedical informatics. Journal of Biomedical Informatics 2014;48:193–204. http://dx.doi.org/10.1016/j.jbi.2014.02.013.
[10] Heinisch O. Cochran,W. G.: Sampling Techniques, 2. Aufl. John Wiley and Sons, New York, London 1963. Preis s. Biometrische Zeitschrift 1965;7(3):203–203. http://dx.doi.org/10.1002/bimj.19650070312.
[11] Liao HS, Akhtar I, Werner C, Slipets R, Pereda J, Wang JH, et al. Open-source controller for low-cost and high-speed atomic force microscopy imaging of skin corneocyte nanotextures. HardwareX 2022;12:e00341. https://doi.org/10.1016/j.ohx.2022.e00341.
[12] E.-T. Hwu, A. Boisen, Hacking CD/DVD/Blu-ray for biosensing. ACS Sens. 2018; 1222–1232, https://doi.org/10.1021/acssensors.8b00340.
[13] Riethmuller C,McAleerMA, Koppes SA, Abdayem R, Franz J, Haftek M, et al. Filaggrin breakdown products determine corneocyte conformation in patients with atopic dermatitis. Journal of Allergy and Clinical Immunology 2015;136:1573–1580.e2. https://doi.org/10.1016%2Fj.jaci.2015.04.042.
